# Novelties in Sport Sciences: Novi Sad, Serbia. 11 September 2021

**DOI:** 10.1186/s12919-021-00227-2

**Published:** 2021-11-30

## A1 Symposium summary

### Patrik Drid^1^, Antonino Bianco^2^, Sergey Tabakov^3^, Ewan Thomas^2^

#### ^1^Faculty of Sport and Physical Education, University of Novi Sad, Novi Sad, Serbia; ^2^Sport and Exercise Sciences Research Unit, University of Palermo, Palermo, Italy; ^3^Russian State University of Physical Education, Sports, Youth and Tourism, Moscow, Russia

##### **Correspondence:** Patrik Drid (patrikdrid@gmail.com)

The international conference organized by Combat Sports Lab which took place in Novi Sad, the 11^th^ of September 2021, captures a program of presentations, panel discussions and interactive dialogue which leads the way in novelties within the field of sport science.

The event brought together 36 distinguished scholars from 7 countries around the world, for a total of 20 scientific contributions. The event hosted by the University of Novi Sad, Faculty of Sport and Physical Education and the International Public Organization for the Promotion of Science and Sports “Sport, People, Health”, Branch office Novi Sad, Serbia has been supported by the Provincial Secretariat for Higher Education and Scientific Research of the Republic of Serbia.

The excellence of this meeting is a credit to every participant, who has contributed to the discussion. Among the many dialogs opened during the “Novelties in Sport Sciences” congress we appreciated several nutritional aspects as the role of nutrition as a treatment for metabolic diseases and diabetes, novel nutritional tools to improve performance through specific targets in the gut microbiota and new supplements for power sports or the use of molecular hydrogen for performance enhancement. Important aspects related to cardiovascular profiles of judo athletes and screening of cardiovascular disease in elite sports have been also taken into account as well as sport bioethics, sport sociology and sport psychology. Last but not least, the congress addressed aspects of innovation within the field of strength and conditioning in sport science with important topics regarding rapid weight loss in combat sports, effects of specific training modes and core stabilization and the role of physical activity in children and adolescence.

Overall, the conference was quite successful. While the congress was rich in new information and novel insights, it also highlighted areas of possible development. These include a better understanding of the role of nutrition in professional sports and important aspects related to strength training in children.

Again we wish to thank all the attendees, presenters, speakers and facilitators for the contents and insights provided. The organization committee and congress participants hope this volume will stimulate further investigations and collaborations for future research.

## A2 Routes for the application of exercise-nutrition integrated approach in metabolic diseases

### Giuseppe D’Antona (giuseppe.dantona@unipv.it)

#### CRIAMS Sport Medicine Centre Voghera, University of Pavia, Pavia, Italy

**Background**

The field of rare diseases opens up the possibility of exploring fundamental aspects of exercise physiology, and to deepen our understanding of the physiological significance of new acquisitions from the bench. This is certainly the case with rare metabolic disorders including those of carbohydrates and fats.

Among the inherited disorders dealing with the fatty acids metabolism, the myopathic form of carnitine palmitoyl transferase II (CPTII) deficiency, first reported in 1973 [1], which is due to missense mutations of the CPT2 gene, may represent a precious prototype to further investigate the fundamental interplay between exercise, nutrition and fat handling by subcellular organelles, including mitochondria and peroxisomes. This disorder affects the mitochondrial transport and oxidation of long-chain fatty acids (LCFA) and represents the least severe form of CPTII deficiency. The clinical presentation includes recurrent bouts of rhabdomyolysis, muscle aches and fatigue, usually arising after prolonged exercise and aggravated by the exposure to extreme temperatures, prolonged fasting and intercurrent viral infections. Episodes of rhabdomyolysis are associated with a significant increase in serum creatine phosphokinase (CPK) and myoglobinuria (75% of cases) and may result in renal failure (in 8-25% of cases). Considering the risk of rabdomyolitic events, rest and a high CHO/low fat diet represent the current treatment of the disease. This conservative approach, firmly linked to the concept of *primum non nocere,* does not take into proper account fundamental aspects of the disease physiopathology, and the role of energy sources in the frame of exercise intensities and/or durations, and their utilization. In particular, it is widely known that FAs are the prevalent fuel source of muscle energy during submaximal exercise intensities (<65% VO2 max), [2], with a maximal FAs utilization (FATmax) occurs between 45 and 65% of VO2max. At higher intensities and shorter durations the contribution of CHO as energy source progressively increases and the use of FAs to maintain energy availability is relatively reduced. Based on this background we recently demontrated safety and efficacy of a high intensity interval training (HIIT) combined with resistance training (RT) in a young woman carrying the most frequent mutation (p.Ser113Leu) leading to CPTII insufficiency [3]. Another aspect that deserves attention regards the current nutritional approach for CPTII deficiency. Fat restriction to 20% of total energy expenditure and CHO increase to 65-70% is generally recommended. This approach has been linked to decreased perceived exertion and increased exercise endurance compared to control diet (60% fats, 15% proteins, and 25% carbohydrates). Anyhow, evidences suggest that this nutrients distribution is unable to effectively limit the recurrence of muscle weakness, myalgia and rhabdomyolysis episodes [4]. Indeed, the long and middle term possible effects of such approach on glucose and fat metabolisms are not adequately addressed. This may include the negative impact of hyperglycemia with hyperinsulinemia on CPTI and CPTII activities that may lead to a decrease in the transport of LCFAs into the mitochondria, triglycerides accumulation, and final development of insulin resistance. This vicious circle may be amplified by concomitant recommended reduction in the level of physical activity.

**Conclusions**

The role of the residual fat oxidation represents another intriguing aspect of CPTII deficiency. It has been shown that the defective LCFA transport into the mitochondria can be highly variable among patients, with a range of residual function from 15 to 50% compared to normal subjects. This evidence may represent the physiological justification on for the patients’ capacity to sustain the daily life activities without the occurrence of muscular symptoms. On the other side, the presence of compensatory mechanisms and the possible interplay between organelles devoted to the fats handling should be taken into consideration. In this context, crossroads between mitochondria and peroxisomes have been recently identified and the peroxisome contribution to the total cellular beta oxydation (in the range between 5%-30%) unveiled. A recent forum of discussion of all these physiological and pathophysiological aspects and their translation to clinical practice has been put forward by our research group in a recently published paper on the topic [5].

**References**

1. DiMauro S, DiMauro PMM. Muscle carnitine palmityltransferase deficiency and myoglobinuria. Science 1973;182:929-931.

2. Purdom T, Kravitz L, Dokladny K, Mermier C. Understanding the factors that effect maximal fat oxidation. J. Int. Soc. Sports Nutr. 2018;15:3.

3. Parimbelli M, Pezzotti E, Negro M, Calanni L, Allemano S, Bernardi M, Berardinelli A, D'Antona G. Nutrition and Exercise in a Case of Carnitine Palmitoyl-Transferase II Deficiency. Front. Physiol. 2021;17;12:637406.

4. Schnedl WJ, Schenk M, Enko D, Mangge H. Severe rhabdomyolysis in homozygote carnitine palmitoyltransferase II deficiency. EXCLI J. 2020;19:1309-1313.

5. Negro M, Cerullo G, Parimbelli M, Ravazzani A, Feletti F, Berardinelli A, Cena H, D'Antona G. Exercise, Nutrition, and Supplements in the Muscle Carnitine Palmitoyl-Transferase II Deficiency: New Theoretical Bases for Potential Applications. Front. Physiol. 2021;2(12):704290.

## A3 Exosomes, exercise and nutrition in diabetes: the APETEX project

### Cristina Casals^1,2^ (cristina.casals@gm.uca.es)

#### ^1^MOVE-IT Research Group, Department of Physical Education, Faculty of Education Sciences, University of Cadiz, Spain; ^2^Biomedical Research and Innovation Institute of Cadiz (INiBICA), University Hospital Puerta del Mar, Spain

**Background**

The prevalence of type 2 diabetes mellitus (T2DM) increases along with obesity, in part, due to lack of time for physical activity. Although physical exercise is a therapeutic tool in T2DM, more efficient strategies of high intensity and short duration exercise are proposed. Physical exercise leads to the release of extravesicles, called exosomes, which act as transmitters of information in those involved in improving insulin sensitivity, among others. In fact, the pathophysiology of obesity-induced insulin resistance is explained by ectopic lipid deposition, inflammation of adipose tissue, and organ-tissue communication through exosomes. The combination of physical exercise and nutritional counselling is known to improve the impact on both body composition and health in general population and in T2DM; however, the adherence of those programs is compromised and even the complete intervention is performed by the patient, we can find in the literature *non-responders*. The underlying mechanisms that determine the impact of educational program focused on healthy lifestyles could be explained by molecular and physiological responses to the interventions. In this sense, hormones that regulate appetite and exerkines could impact on food preference, decision making, and in the exercise impact on appetite, consequently, modifying dietary intake, body composition and health. Thus, the main objective of APETEX project is to determine the effects of two 12-week physical exercise programs (HIIT vs. MICT) and a nutritional counselling intervention on the profile of exosomes and to determine their influence on the regulation of appetite, control of insulin sensitivity, body composition and fat metabolism in obese people with T2DM from 40 to 55 years old and of both sexes.

**Methods**

The project is a randomized controlled clinical trial composed of 120 participants, the design has two 12-week interventions; the main factor has 2 levels: participants who receive the nutritional education (EDU) and controls (CG); the second factor has 3 levels: high-intensity interval training (HIIT), moderate-intensity continuous training (MICT), and controls (INACT). Therefore, participants will be randomized into 6 groups (n=20), adjusted by gender ~50% in each group: EDU+HIIT, EDU+MICT, EDU+INACT, CG+HIIT, CG+MICT, CG+INACT. The interaction between factors and independent effects will be analysed. The evaluation will be performed pre and post the 12-week intervention consisted in nutritional assessment (body composition and nutritional habits), insulin resistance (HOMA), exosomes profile, appetite regulation hormones, fat oxidation capacity during exercise, physical activity habits, and quality of life. As a main outcome, the food preference will be assessed through electroencephalogram (EEG) showing to the participant, in fasting condition, different types and caloric density food. In summary, further comprehensive approaches to establish causes and underlying mechanisms of T2DM are needed, and education interventions should be optimized to avoid failure. The multidisciplinary team consisted of physicians, physical activity experts, biologist, psychologist and nutritionist researchers with experience on education programs and the outcomes of this project, and the availability of structure to execute the proposed design the support of primary health-care centres, hospital and sports centres of councils, ensure a great viability.

**Results**

In this sense, the appetite regulation through exosomes and its impact on body composition and health could be a main factor in the efficacy of education programs aimed at change lifestyles of adult people. Moreover, many studies have investigated diet and physical exercise influence, but few have investigated how exosomes regulates appetite and metabolism, directly related with the development of T2DM, being necessary a gender analysis. This field of research, from multidisciplinary approaches, would have high economic and social impact in a population in which the prevalence of T2DM is increasing every year worldwide due to physical inactivity and unhealthy dietary patterns. Moreover, these results will be combined with those of Professor Jesús G. Ponce-Gonzalez with the EDUGUTION project (PID2019-110063RA-I00) and LIPIDOX (LI19/21IN-CO09) in which gut microbiota will be analysed, allowing the study of the gut-brain axis, and, through muscle biopsies, signalling cascades of insulin, fat oxidation and mitochondrial biogenesis will be evaluated. Consequently, the results of these projects developed at the University of Cadiz by the MOVE-IT Research group will be of interest both nationally and internationally for the prevention of T2DM and, when not possible, to minimize the deterioration on health and quality of life that this chronic disease implies.

**Conclusions**

According to the WHO, diabetes causes 3.2 million deaths per year all over the world and approximately 500 million people have T2DM and it is increasing year by year. The complications of T2DM also impact on health costs derived from diabetes, such as heart attack, stroke, blindness, kidney failure and lower limb amputation. According to the 2016 Global Report on Diabetes, T2DM and its complications are largely preventable through adoption of policies to create supportive environments for healthy lifestyles, including the behavioural change caused by education programs.

**Funding**

The present research project is entitled “Efficacy of a nutritional education strategy and role of physical exercise on the regulation of appetite and body composition through the profile of exosomes in type 2 diabetics (The APETEX project),” code PID2020-120034RA-I00, funded by the Ministry of Science and Innovation of Spain, from 2021 to 2024.

## A4 Precision nutrition tools for improved athletic performance - power of nutrigenetics and gut microbiota in a future of sport nutrition

### Manja M. Zec (manjazec@email.arizona.edu)

#### School of Nutritional Sciences and Wellness, University of Arizona, Tucson 85721, AZ, USA

**Background**

In professional athletes with high nutritional and caloric requirements and regular bouts of exercise followed by immuno-metabolic adaptations, the necessity for specific clinical and nutritional guidelines is evident. Precision nutrition approach for improved individual sport performance and maintaining overall fitness and wellness is a forthcoming strategy in dietary and nutritional recommendations in those subjects. This narrative review briefly summarizes current highlights on nutrigenetics and gut microbiota in elite-level athletes, conferring the role for evidence-based precision nutrition advice for: (a) maximization of sport performance and preventing its impairment (b) injury prevention (c) disease risk alleviation. Genetic and gut microbiota profiles from elite athletes might inform about an individual's (a) burden, i.e., health or genetic risk (b) and/or selective advantage in terms of maximizing sport performance. Both genetic makeup and gut microbial profiles interact with environmental factors, markedly nutrition, which influences final phenotype including sport performance. Precision nutrition "*accounts for interindividual differences*" [1], and proper application of precision nutrition advice translates personalized predispositions to an individual's selective advantage. Herein are outlined certain nutrigenetic and gut microbiota candidate biomarkers with a potentially powerful role in customizing actionable nutritional and dietary advice for improved sport performance.

***The Aspect of Nutrigenetics.*** Recent review summarized candidate genes potentially influencing performance, wellness, and general health in elite athletes [2], such as genes mediating caffeine metabolism investigated for ergogenic potential; then genes involved in essential micronutrient metabolism, especially role of vitamin D and calcium in bone and muscle turnover; genes involved in biochemical pathways providing carbon fuel in the human body, like *MTHFR* in folate metabolism. Furthermore, genes which protein products participate macronutrient processing, such as *FADS* genes with ethnic-specific distributions, inform the susceptibility to omega-3 deficiency syndrome associated with various chronic diseases. Given the chronic low-inflammatory burden, the athletes are exposed to, as a metabolic adaptation in muscles, variability in *IL-6* and *TNF-α* might influence intrinsic inflammatory defense. Further, the rs1558902 *FTO* site is protective from obesity, with prominent effects on a high-protein diet, an example of a gene-dietary interaction. Also, in resistance-trained subjects, caffeine demonstrated the greatest ergogenic effects when administered an hour before training or an hour before a peak is anticipated in lower-body muscular performance [3], imposing the role for the timing of supplementation. Genetic variations and interactions with nutrients and environmental parameters result in distinctive metabotypes and phenotypes, which might further serve as classification parameters for allocating athletes to a specific group according to the level and type of dietary intervention needed for their maximal output. Given thousands of genes in the human genome, as well as their multiple interactions, the need for a mosaic, multidisciplinary and multifactorial research to be undertaken for the advancement of sport nutrition supported by nutrigenetic information is evident. In the future, a shortlisted set of the most penetrable genes, and those taking part in polygenic traits and orchestrating the most prominent features for sport performance maximizations, should be established to form part of regular genetic and clinical testing of an athlete.

***The Aspect of Gut Microbiota Diversity*.** A type of sport followed by dietary profiles induces differences in gut microbiota. Exercise typically increases some probiotic and gut integrity conforming taxa, *Bifidobacterium*, *Lactobacilli*, and *Akkermansia* [1]. Low FODMAP dietary pattern, although protective of a sensitive gut, gastrointestinal bloating, and other distress, nevertheless might limit carbohydrate components with beneficial prebiotic effects on the intestinal microbiota, and endurance sports commonly require high-carbohydrate diets. Furthermore, athletes exert decreased levels of LPS polysaccharide originating from the gram-negative bacteria cell wall and leading to increased gut permeability, gut leaking, and induced proinflammatory condition – altogether advocating for increased gut integrity and distinctive gut microbial features in the professionals. Exercise induces favourable gut microbiota variability linked with augmented performance, although excessive training must be monitored in terms of the possible dehydration and even ischemia of the gut, linked with impaired integrity and gastrointestinal discomfort. Athletes commonly have higher microbiota abundance, as demonstrated in professional rugby players in comparison with controls [4] and reviewed in [5]. A study in four male trans-oceanic rowers showed a favourable increase in microbial diversity upon the 33-day event in terms of enriched butyrate-producing species and those linked with improved insulin-sensitivity, with the changes remaining stable upon three-months. In female rowers, Firmicutes/Bacteroidetes ratio was higher in the elite vs. a non-elite group. In healthy young adults, the ratio was directly associated with VO2 max levels but not nutritional and anthropometric indicators, pointing out its relevance in mediating physiological response to exercise. Interestingly, Firmicutes/Bacteroidetes ratio is generally implicated in obesity, and Bacteroidetes exert the role in the metabolic processing of complex sugars and degradation of proteins.

***The Aspect of Gut Microbiota Functional Capacity*.** Intestinal microbiota essentially contributes to immunological and inflammatory defence by actively participating in macronutrient break-down, absorption, and further utilization by various organs, including skeletal muscle [1]. Muscles undergo an extensive post-training repair process, in addition to anti-inflammatory defense, due to the increased energy expenditure and protein degradation. The gut-muscle axis provides important molecular fuel during exercise, especially in terms of adequate protein supply. Carbohydrate metabolism linked with the adequate levels of short-chain fatty acids (SCFA) from dietary fibers generally beneficially impacts insulin regulation and partly mediates desirable protein synthesis and absorption. Still, fiber intake might induce bloating and gastrointestinal discomfort and should be timed properly in professional athletes. Furthermore, dietary supply of proteins provides sufficient levels of branched-chain amino acids, carnitine, choline, etc., which aid in sustaining necessary muscle structure and function, however proteolytic gut microbial products such as trimethylamine-N-oxide deteriorate gut integrity, potentially leading to gastrointestinal distress and inflammation [1]. Hence, in professional athletes with almost as double as regular daily protein requirements, the proper saccharolytic-to-proteolytic gut microbial function should be actively maintained by modes of precision nutrition. The SCFA are produced by fermentation of undigested plant fibers in the large intestine and are associated with insulin sensitivity, diabetic improvements, and other cardio metabolic benefits. Sport performance might be supported by both butyrate and propionate, the latter potentially derived through lactate conversion in a post-exercise setting, mediated by bacterial genus *Veillonella*, as indicated in marathon-runners, ultra-marathoners, and rowers at the Olympics [6]. Another study [7] in amateur runners demonstrated an increase in *Coriobacteriaceae* microbial family after the run, the taxa involved in steroid and bile salts metabolism, as well as metabolism of polyphenol products. Given the issue with polyphenol availability, their versatile health-improving properties might be augmented with exercise-induced increase in *Coriobacteriaceae* involved in the polyphenol breakdown. This is an illustrative example of the concept on which precision nutrition should rely.

***Interaction effects of nutrigenetic and gut microbiota approach*.** Long-chain polyunsaturated fat (PUFA) metabolism reflects fat intake and is also genetically determined. Furthermore, PUFA intake influences essential inflammatory balance by modulating gut bacterial profiles, with higher omega-3 PUFA/saturated fats ratios promoting more favourable profiles. Another example is lactose, which in addition to fructose, glucose, sucrose, or dextrose, might serve as a simple sugar, carbohydrate fuel, necessary as a primary energy source during bouts of exercise. Although lactose sugar increases probiotic *Bifidobacteria* and *Lactobacilli* levels, thus supporting gut eubiosis, there is a percentage of lactose-intolerant subjects in a general population due to a single genetic variation, for whom this type of energy substitution would not work. The nutrigenetic-gut microbiota axis illustrates the complexity of precision nutrition strategies, as well as intertwining relationships between different components of a personalized approach.

**Conclusions**

Future of sport nutrition urges for a holistic, fast-forward, decision-making process based on a comprehensive set of properly integrated information pertaining to an athlete's individual predispositions, like genetic and gut microbial risk factors. Research in precision nutrition is rather young, and more well-powered studies are needed to confirm particular risk factors in athletes and stratify responders from non-responders in the presence of a certain nutrient or food bioactive, the food itself, or a diet. Precision nutrition algorithms in professional athletes should be built upon a transdisciplinary approach, encompassing orchestrated input from molecular biologists, clinical nutritionists, data scientists, and sport physiologists, and finally articulated through a recommendation by a team of dietitians practicing in athletes. Future sport science endeavours should tackle the unexplained variability in our understanding of gene effects as well as microbiota functions in terms of sport performance maximization, which should be interpreted in the context of a panel of blood, physiological, and physique determinants. Finally, a proper design of precision nutrition solutions must account for sport features itself, intensity, duration, and frequency of training.

**References**

1. Hughes RL, Holscher HD. Fueling Gut Microbes: A Review of the Interaction between Diet, Exercise, and the Gut Microbiota in Athletes. Adv. Nutr. 2021.

2. Guest NS, Horne J, Vanderhout SM, El-Sohemy A. Sport Nutrigenomics: Personalized Nutrition for Athletic Performance. Front. Nutr. 2019;6:8.

3. Harty PS, Zabriskie HA, Stecker RA, Currier BS, Tinsley GM, Surowiec K, et al. Caffeine Timing Improves Lower-Body Muscular Performance: A Randomized Trial. Front. Nutr. 2020;7:585900.

4. Clarke SF, Murphy EF, O'Sullivan O, Lucey AJ, Humphreys M, Hogan A, et al. Exercise and associated dietary extremes impact on gut microbial diversity. Gut. 2014;63(12):1913-1920.

5. Mohr AE, Jager R, Carpenter KC, Kerksick CM, Purpura M, Townsend JR, et al. The athletic gut microbiota. J. Int. Soc. Sports Nutr. 2020;17(1):24.

6. Scheiman J, Luber JM, Chavkin TA, MacDonald T, Tung A, Pham LD, et al. Meta-omics analysis of elite athletes identifies a performance-enhancing microbe that functions via lactate metabolism. Nat. Med. 2019;25(7):1104-1109.

7. Zhao X, Zhang Z, Hu B, Huang W, Yuan C, Zou L. Response of Gut Microbiota to Metabolite Changes Induced by Endurance Exercise. Front. Microbiol. 2018;9:765.

## A5 Guanidinoacetic acid as a novel supplement in power sports

### Valdemar Štajer, Nikola Todorović, Jovan Kuzmanović, Marijana Ranisavljev, Dara Korovljev, Sergej Ostojić

#### Faculty of Sport and Physical Education, University of Novi Sad, Novi Sad, Serbia

##### **Correspondence:** Valdemar Štajer (stajervaldemar@yahoo.com)

**Background**

Guanidinoacetic acid (GAA) is a naturally occurring amino acid derivate that acts as a direct precursor of creatine, an essential contributor to cellular bioenergetics of muscle and nerve tissue. GAA is formed endogenously in our bodies through the enzyme-catalyzed step process from arginine and glycine, mainly in the kidney and pancreas. Although it plays a key role in creatine synthesis, metabolism GAA is only recently recognized as a sport-enhancing agent, and it can be also used as a novel biomarker of fatigue. Up to date, only a few studies evaluated the safety and efficiency of GAA. Further, there are special indications for the effectiveness of the use of this supplement in power sports athletes, especially in combat sports. Combat sports require high levels of metabolic conditioning. The purpose of this mini-review is to critically analyzed current literature concerning GAA supplementation, evaluate GAA safety, effectiveness, and application in power sports.

**Methods**

Research on available literature was conducted on PubMed (Medline) database. All studies that include GAA supplementation effects on exercise and sport performance were considered for inclusion in the study.

**Results**

Following the literature review, we found 77 studies that included the keywords: "GAA" AND "exercise"; "GAA" AND "sport"; "GAA" AND "sport performance"; "GAA" AND "safety." After reading the abstract or full text, six studies were included in the review. The evidence shows that there justified presumption that GAA can be considered as an effective performance-enhancing agent. GAA improves muscle strength, while on the other side it can reduce fatigue. GAA has more ways of entering the cell than creatine, and it seems that it can improve and have a more significant effect in muscle groups with lower initial strength levels. This can be extremely important in sport with specific upper body strength and power demands. Also, GAA does not produce water retention, which is an important factor in combat sports due to rapid weight loss practices. In terms of safety, there is a need for quality safety studies, but based on the evidence so far, it seems that the combination of GAA with creatine or B vitamin can be considered safe to use. The results are represented in Table 1.

**Conclusion**

Based on the available data, GAA can be considered as a novel, highly effective supplement, especially in power sport athletes. Therefore, future studies should be based on determining adequate supplementation protocols, maximizing the effects of supplementation, and examining safety during longer-term consumption periods.

**References**

1. Ostojic SM, Stojanovic MD, Hoffman JR. Six-week oral guanidinoacetic acid administration improves muscular performance in healthy volunteers. J. Investig. Med. 2015;63(8):942-946.

2. Semeredi S, Stajer V, Ostojic J, Vranes M, Ostojic SM. Guanidinoacetic acid with creatine compared with creatine alone for tissue creatine content, hyperhomocysteinemia, and exercise performance: A randomized, double-blind superiority trial. Nutrition 2019;57:162-166.

3. Ostojic SM, Niess B, Stojanovic M, Obrenovic M. Creatine metabolism and safety profiles after six-week oral guanidinoacetic acid administration in healthy humans. Int. J. Med. Sci. 2013;10(2):141.

4. Ostojic SM, Niess B, Stojanovic M, Obrenovic M. Co-administration of methyl donors along with guanidinoacetic acid reduces the incidence of hyperhomocysteinaemia compared with guanidinoacetic acid administration alone. Br. J. Nutr. 2013;110(5):865-870.

5. Ostojic SM, Stojanovic M, Drid P, Hoffman JR. Dose-response effects of oral guanidinoacetic acid on serum creatine, homocysteine and B vitamins levels. Eur. J. Nutr. 2014;53(8):1637-1643.

6. Ostojic SM, Todorovic N, Stajer V. Effect of Creatine and Guanidinoacetate Supplementation on Plasma Homocysteine in Metabolically Healthy Men and Women. Ann. Nutr. Metab. 2021;17:1-2.

**Table 1 (abstract A5).** Study characteristics

Study	N	Study design	Supplementation protocols	Outcome
Ostojic et al. [1]	48	RCT	GAA (1.2 ^**a**^, 2.4 ^**b**^, or 4.8 ^**c**^ gr/d) or placebo ^**d**^ for 6 weeks	Handgrip strength ↑ ^**a, b, c**^ 1 RM Bench press ↑ ^**a, b, c**^ Vertical jump ↔ ^**a, b, c, d**^ Anaerobic power ↔ ^**a, b, c, d**^ Aerobic capacity ↔ ^**a, b, c, d**^
Semeredi et al. [2]	14	RCCT	1 gr of GAA and 3 gr of creatine/d ^**a**^ or 4 gr/d of creatine for 4 weeks ^**b**^	Total creatine ↑ ^**a, b**^ Brain creatine ↑ ^**a, b**^ 1RM Bench press ↑ ^**a, b**^ tHcy (μmol/L) ↔ ^**a**^ VO2max ↔ ^**a, b**^
Ostojic et al. [3]	24	RCT	GAA (2.4 gr/d) ^**a**^ or placebo ^**b**^ by oral administration for 6 weeks	Serum GAA ↑ ^**a**^ Serum creatine ↑ ^**a**^ Serum creatinine ↑ ^**a**^ Serum tHcy ↑ ^**a**^ Serum AST↔ ^**a, b**^
Ostojic et al. [4]	24	RCT	2·4 gr GAA/d ^**a**^, or 2·4 gr/d of GAA with 1·6 gr/d of betaine hydrochloride, 5 mg/d of vitamin B12, 10 mg/d of vitamin B6, and 600 mg/d of folic acid ^**b**^	Serum creatine ↑ ^**a, b**^ Serum tHcy ↑ ^**a**^, ↔ ^**b**^
Ostojic et al. [5]	48	RCT	GAA (1.2 ^**a**^, 2.4 ^**b**^, or 4.8 ^**c**^ gr/d) or placebo (Inulin) ^**d**^ for 6 weeks	Serum GAA **↑** ^**a, b, c**^ Serum creatine **↑** ^**a, b, c**^ Serum creatinine **↑** ^**a, b, c**^ Vitamin B6, B12 **↔** ^**a, b, c, d**^ tHcy (μmol/L) **↔** ^**a, b, d**^**; ↑** ^**c**^
Ostojic et al. [6]	n=10	UINT	2 gr of GAA and 2 gr of creatine/d for 4 weeks	tHcy (μmol/L) **↑**

**Legend**: N – participants, RCT- Randomized, double-blind control trail, RCCT- Randomized, double-blind crossover trail, UINT - Uncontrolled interventional trial, GAA - Guanidinoacetic acid, tHcy- total homocysteine; ↑- increased; ↔ no change; gr/d- grams per day; Asterix ^**a, b, c, d**^ - represents different study groups

## A6 Metabolic syndrome or health after professional sport

### Tatjana Trivić (ttrivic@yahoo.com)

#### Faculty of Sport and Physical Education, University of Novi Sad, Novi Sad, Serbia

**Background**

The effects of a physically active lifestyle on former athletes' health have become a rapidly advancing area of research. Many lifestyle factors have been associated with metabolic syndrome (MetS). However, most of these studies have not considered the potential impact MetS on former athlete's health. Therefore, this study could provide another point of view on aspects related to MetS and former athletes with respect to multiple lifestyle factors. Exercise and physical activity are associated with better quality of life and health outcomes. Still, there are also negative aspects of sports that are not noticeable during active participation in sports. For these reasons, the relation between physical exercise and former athlete's health has increased significantly over recent years. Former athletes represent a distinct group of individuals who have exercised for several years and regularly participated in competitions different from the general population during their lifetime. Metabolic syndrome is characterized by the presence of any 3 of 5 defined abnormalities: elevated waist circumference, blood triglycerides, blood pressure or fasting glucose, and reduced blood HDL cholesterol. It's defined by a combination of different physiological, biochemical, clinical, and metabolic factors that directly increases the risk of atherosclerotic cardiovascular disease, type 2 diabetes mellitus, and all-cause mortality [1]. However, according to previous studies, former athletes have a significantly lower risk for disturbances in glucose regulation and coronary heart disease than controls. In addition, former athletes had a lower risk for MetS and better metabolic health in late life [2]. Some studies suggest that former athletes have a lower prevalence of cardiovascular disease, hypertension, and diabetes due to their sport careers. In contrast, others claim that is only the consequence of being more physically active in later life compared to non-athletes. The purpose of this work is to investigate existing literature about the prevalence of MetS and its components in former athletes.

**Methods**

Comparison of various studies about prevalence of MetS and its components in former athletes.

**Results**

Some studies indicate that former athletes had a significantly lower incidence of type 2 diabetes, high blood pressure, metabolic syndrome, sitting time, and physical inactivity. On the contrary, the prevalence of MetS is observed among former endurance, football, and mixed-sports players. Also, in former athletes of power sports higher average amount of total body weight, body mass index when compared with active athletes and non-athletes were noted. Moreover, former athletes who maintained their physical activity at recommended levels remained less likely to have MetS. The metabolic syndrome is highly prevalent in populations around the world, regardless of the definition used. Physical inactivity and obesity are two of the major modifiable risk factors for metabolic syndrome. It is well known that lifestyle plays a vital role in the preventable risk factors that underlie most chronic diseases as well in the MetS, in particular. The skeletal muscle is the most insulin-sensitive tissue in the body. Therefore, physical activity has been shown to reduce skeletal muscle lipid levels and insulin resistance, regardless of BMI [3]. Research has shown that the prevalence of MetS, increases among former athletes [4]. It has been reported that many former athletes adopt a sedentary lifestyle after their retirement, which can cause a threat of MetS to their health [5]. On the contrary, former athletes tend to keep their fitness advantage over non-athletes and have healthier habits.

**Conclusions**

It is necessary for former athletes to adopt healthier lifestyles after their career, which may give them an advantage regarding the health risk factors and prevent metabolic syndrome. Furthermore, physical activity at recommended levels seems to play an important role in the association with the metabolic syndrome of former athletes. Former athletes diagnosed with metabolic syndrome should be encouraged to change their diet and exercise habits as primary therapy. It seems that lifestyle plays a vital role in former athletes that underlie the MetS. So, former athletes should be encouraged to focus on maintaining and improving their personal level of physical activity.

**References**

1. Kaur J. A comprehensive review on metabolic syndrome. Cardiol. Res. Pract. 2014;4301528.

2. Laine MK, Eriksson JG, Kujala UM, Kaprio J, Loo BM, Sundvall J, Bäckmand HM, Peltonen M, Jula A, Sarna, S. Former male elite athletes have better metabolic health in late life than their controls. Scand. J. Med. Sci. Sports 2016;26(3):284-290.

3. Goodpaster BH, He J, Watkins S, Kelley DE. Skeletal muscle lipid content and insulin resistance: evidence for a paradox in endurance-trained athletes. J. Clin. Endocrinol. Metab. 2001;86(12):5755-5761.

4. Emami M, Behforouz A, Jarahi L, Zarifian A, Rashidlamir A, Rashed MM, Khaleghzade H, Ghaneifar Z, Safarian M, Azimi-Nezhad M, Nikroo H**,** Nematy, M. The risk of developing obesity, insulin resistance, and metabolic syndrome in former power-sports athletes-Does sports career termination increase the risk. Indian. J. Endocrinol. Metab. 2018;22(4):515-519.

5. Panayiotoglou A, Grammatikopoulou MG, Maraki MI, Chourdakis M, Gkiouras K, Theodoridis X, Papadopoulou SK, Famisis K, Hassapidou, M. N. Metabolic syndrome in retired soccer players: A pilot study. Obes. Med. 2017;8:15-22.

## A7 Screening for cardiovascular diseases in athletes

### Aleksandra Milovančev (aleksandra.milovancev@mf.uns.ac.rs)

#### Faculty of Medicine, University of Novi Sad, Novi Sad, Serbia

**Background**

Long term athletic training is associated with a sequence of adaptations in cardiac structure, function, and electrical activity, these alterations are collectively termed athlete’s heart [1]. Athlete’s heart is a normal, physiological adaptation of the body to the intensive workout and aerobic exercise. Heart adapts with ventricular wall thickening and chamber enlargement. As a result geometry of the heart is changed. The magnitude of cardiac remodelling depends on the sport discipline practiced. Endurance sports make the biggest impact on heart geometry changes [2]. For example Wrestling/Judo are characterized with high isometric exercise but make moderate cardiac remodelling. The athletes level of expertise and the number of hours training make a big difference when cardiac remodelling is in question even among similar sport disciplines. Cardiovascular (CV) screening in athletes is widely recommended and routinely performed before participation in competitive sports [3]. There is general agreement that early detection of cardiac conditions at risk for sudden cardiac death (SCD) is an important objective, but the optimal strategy for CV screening in athletes remains an issue of considerable debate. Exercise is a known trigger and can unmask occult cardiac disease to precipitate SCD. The primary goal of CV screening of athletes is to identify underlying cardiac disorders predisposing to SCD. The current pre-participation history and physical examination, while pragmatic and widely practiced, is limited in its ability to identify athletes with conditions at risk for SCD. The current model uses a comprehensive history questionnaire and physical examination. The electrocardiogram (ECG) increases early detection of some cardiac disorders associated with SCD. Electrocardiogram interpretation accuracy and reliability are challenging. In choosing a screening strategy, sports medicine physicians approach should take an individual risk of the athlete, physician expertise and available cardiology resources for accurate ECG interpretation and the secondary evaluation of ECG abnormalities, as well as their assessment that a particular screening strategy that will provide more benefit than harm. SCD prevalence is low [4], but when it happens it is devastating. The most commonly reported causes of SCD in athletes include hypertrophic cardiomyopathy (HCM), anomalous coronary arteries, idiopathic left ventricular hypertrophy, arrhythmogenic right ventricular cardiomyopathy, dilated cardiomyopathy, myocarditis, long QT syndrome (LQTS), ventricular pre excitation/Wolff–Parkinson–White (WPW), aortic dissection, and atherosclerotic coronary artery disease (CAD). In 44% of SCD no structural cardiac abnormalities were seen on autopsy this may be due to primary electrical diseases and inherited arrhythmia syndromes. An estimated 60% of the disorders associated with SCD in young individuals may have detectable ECG abnormalities. The initial cardiovascular protocol includes family and personal history, physical examination with determination of blood pressure, and basal 12-lead ECG. Additional tests, such as echocardiography, 24-h Holter monitoring, or stress testing, SAECG, and cardiac MRI are requested only for subjects who had positive findings at the initial evaluation. In uncertain cases, invasive tests such as contrast ventriculography (both right and left), coronary angiography, endomyocardial biopsy, and electrophysiological study may be necessary in order to confirm (or rule out) the diagnosis of cardiovascular disease [5]. Athletes diagnosed with clinically relevant cardiovascular abnormalities are managed according to available guidelines for assessing athletic risk.

**Conclusions**

Individual approach is needed in every single individual athlete. Intense screening with cardiovascular imaging is indicated in particular cases. Screening procedures during regular training routines are needed. All known strategies for reducing SCD in athletes should be applied in every day practice of sport medical doctors.

**References**

1. Finocchiaro G, Dhutia H, D’Silva A, Malhotra A, Steriotis A, Millar L, et al. Effect of Sex and Sporting Discipline on LV Adaptation to Exercise. JACC Cardiovasc. Imaging. 2017;10(9):965-972.

2. Pelliccia A, Caselli S, Sharma S, Basso C, Bax JJ, Corrado D, et al. European Association of Preventive Cardiology (EAPC) and European Association of Cardiovascular Imaging (EACVI) joint position statement: recommendations for the indication and interpretation of cardiovascular imaging in the evaluation of the athlete’s heart. Eur. Heart. J. 2018;39(21):1949-1969.

3. Drezner JA, O’Connor FG, Harmon KG, Fields KB, Asplund CA, Asif IM, et al. AMSSM Position Statement on Cardiovascular Preparticipation Screening in Athletes: Current Evidence, Knowledge Gaps, Recommendations, and Future Directions. Clin. J. Sport. Med. 2016;26(5):347-361.

4. Takiguchi M, Knight T, Nguyen TT, Limm B, Hayes D, Reddy V, et al. Underdiagnosis of Conditions Associated with Sudden Cardiac Death in Children - Is it the Absence of a Comprehensive Screening Program or a True Low Prevalence? Hawaii J. Med. Public. Health 2016;75(2):42-45.

5. Pelliccia A, Sharma S. The 2020 ESC Guidelines on Sport Cardiology. Eur. Heart. J. 2021;42(1):5-6.

## A8 Cardiac profile of elite judo athletes

### Antonino Bianco^1^, Nemanja Lakićević^1^, Roberto Roklicer^2^, Tatjana Trivić^2^, Aleksandra Milovančev^3^, Giuseppe D’Antona^4^ and Patrik Drid^2^

#### ^1^Sport and Exercise Sciences Research Unit, University of Palermo, Palermo, Italy; ^2^Faculty of Sport and Physical Education, University of Novi Sad, Novi Sad, Serbia; ^3^Faculty of Medicine, University of Novi Sad, Novi Sad, Serbia; ^4^CRIAMS Sport Medicine Centre Voghera, University of Pavia, Pavia, Italy

##### **Correspondence:** Antonino Bianco (antonino.bianco@unipa.it)

**Background**

Being physically active is widely accepted as an effective way to promote health and prevent an incredible spectrum of health risk factors within all age, gender, ethnic and socioeconomic subgroups. Elite athletes have to regularly engage in vigorous physical activity (PA) to gain a competitive advantage over their opponents and excel in their career trajectories. Likewise, carefully designed training has to be followed by adequate dietary intake and recovery to keep an excellent physical shape and prevent overtraining syndrome occurrence. Even though there is a positive relationship between levels of PA and health benefits, especially in terms of its cardioprotective properties [1], elite athletes commonly engage in PA doses that can have pathogenic consequences on various body systems. In the case of the present study, a growing body of evidence shows that judo is a highly demanding type of PA that requires the involvement of the entire body and a modifiable set of technical-tactical skills [2]. In addition, judo athletes must maintain excellent body physique since judo is a weight categorized sport. Therefore, potential health benefits elicited by judo training are dictated in part by a number of training variables such as intensity, frequency, and volume that are inherent to the practice of judo and related factors such as nutrition and rest. Available data indicates that regular, intense PA is associated with a modest increase in left ventricular wall thickness and cavity size, but the extent of these physiological changes is predominantly determined by a variety of demographic factors, which include age, gender, size, ethnicity, and sporting discipline with a minor number of athletes involved in intense isotonic and isometric exercise may exhibit substantial increases in cardiac size that overlap with the epigenetics of the cardiomyopathies [3]. Therefore, the present study aimed to determine whether elite-level judo causes features in heart muscle that deviate substantially from the non-active controls. Our study placed particular emphasis on left ventricular mass (LVM) and left ventricular index (LVMI) athletes and compared these findings to controls of identical body shapes.

**Methods**

Six elite male judokas (mean age: 26.66±2.06 yrs.; mean height 182.83±8.44 cm; mean body weight 85.15±12.21 kg; average weekly exercise volume was 25.0±5.7 hours) and six healthy non-athletes (males) (mean age: 25.50±2.88; mean height 183.33±6.50 cm; mean body weight 80.50±14.58 kg) participated in the study. Relative wall thickness (RWT), left LVM, and LVMI were measured to identify four cardiac profiles among judokas and controls: 1 – normal, 2 – concentric remodelling, 3 – concentric hypertrophy, 4 – eccentric hypertrophy. The study was conducted according to the Helsinki declaration, and ethical approval was obtained from the ethics committee of the University of Novi Sad, Serbia (Ref. No. 46–06-02/2020–1). Statistical analysis was conducted using IBM SPSS Statistics for Windows, Version 20.0. (IBM Corp. 20, Armonk, NY).

**Results**

No significant differences were observed between judokas and the control group in terms of age, body height, and body weight (p>0.05). Relative wall thickness among judokas and controls was also insignificantly different. However, the values of LVM were significantly greater (p=0.001) in judokas compared to the control group 230.99±45.04 vs. 137.16±19.17, respectively. Accordingly, LVMI among judokas was significantly higher (p=0.000) than in controls as well, 111.33±16.41 vs. 67.65±5.81, respectively (Table 1). Of the examined athletes, four judokas belonged to the eccentric hypertrophy group (66.7%), while two participants had normal cardiac remodelling, thus belonging to group one (33.3%).

This study shows LVM and LVMI values were significantly higher in judokas compared to the control group, with four judokas showing eccentric hypertrophy and two judokas expecting heart remodelling. Similar findings were retrieved in the previously published topic of similar nature. For instance, Date et al. [4] found that LVM and LV wall thickness (LVWT) of marathon runners and judo practitioners were significantly greater than those of controls (LVM 318.3 ± 48 vs. 496.2 ± 114.8 vs. 189.7 ± 36 g respectively p<0.0001; LVWT 21.9 ± 1.9vs. 25.5 ± 3.5 vs. 16.2 ± 2.7 mm, p<0.001). The LV end-diastolic dimension index was more significant in the marathon group and more minor in the judokas compared to the control (31.2 ± 2.1 vs. 22.7 ± 2.9 vs. 26.9 ± 2.2 mm/m2). Plasma brain Natriuretic Peptide (NP) concentrations were higher in the judo and marathon groups than in controls and positively correlated with LVM. Sun et al. [5] studied 13 male judokas, which all had increased LVM and LVMI, associated with an increased left ventricular internal diameter (LVIDd). In 6 (46%) of males, LVIDd was above 60mm with LVM ≥274g. In female judokas (n=7), left ventricular (LV) geometry was normal (LVMI 74.7 ± 13.3g/m2) with increased LVIDd (58mm) and LVM (224g) in only one. Whyte et al. [6] noted that judokas exhibited significantly increased LV wall thickness and LVM (males 264 vs. 191g, females LVM 192 vs.132g.) with normal LVIDd and preserved systolic and diastolic function compared to sex-matched controls. Although significantly increased, wall thickness measures were within reference values for all judo athletes. Whyte et al. [7] found that all athletes included in their study had normal diastolic and systolic functions. In 22 male judo athletes, the mean interventricular septal wall thickness was (IVSd) 10.9 mm, mean posterior wall thickness (PWd) was 10.4 mm, and LVM was 259 ± 64 g. In 20 female judokas mean IVSd was 9mm, PWd 8.9 mm and LVM 190 ± 38g. The LVM and LWT of marathon runners and judo athletes were significantly greater than those of controls. Two male judokas had an MWT of 14 mm and LVM of 370, 375g, respectively.

**Conclusions**

Findings of our study indicated that LVM and LVMI values were significantly greater in judo athletes compared to the control group, with four judo athletes displaying eccentric hypertrophy and two judo athletes had expected cardiac remodelling.

**References**

1. Pandey A, Garg S, Khunger M, Darden D, Ayers C, Kumbhani DJ, et al. Dose-Response Relationship Between Physical Activity and Risk of Heart Failure: A Meta-Analysis. Circulation 2015;132:1786-1794.

2. Franchini E, Brito CJ, Fukuda DH, Artioli GG. The physiology of judo-specific training modalities. J. Strength Cond. Res. 2014;28(5):1474-1481.

3. Rawlins J, Bhan A, Sharma S. Left ventricular hypertrophy in athletes. Eur. J. Echocardiogr. 2009;10(3):350-356.

4. Date H, Imamura T, Onitsuka H, Maeno M, Watanabe R, Nishihira K, et al. Differential increase in natriuretic peptides in elite dynamic and static athletes. Circ. J. 2003;67:691-696.

5. Sun B, Ma JZ, Yong YH, Lv YY. The upper limit of physiological cardiac hypertrophy in elite male and female athletes in China. Eur. J. Appl. Physiol. 2007;101:457-463.

6. Whyte G, George K, Sharma S, Martin L, Draper N, McKenna W. Left Ventricular Structure and Function in Elite Judo Players. Clin. Exerc. Physiol. 2000;2:204-208.

7. Whyte GP, George K, Sharma S, Firoozi S, Stephens N, Senior R, et al. The upper limit of physiological cardiac hypertrophy in elite male and female athletes: The British experience. Eur. J. Appl. Physiol. 2004;92:592-597.

**Table 1 (abstract A8).** Demographic characteristics and differences in left ventricular mass and mass index between judokas and the control group

	Judo	Control	F	p	Eta
Age (yrs.)	26.66(2.06)	25.50(2.88)	0.013	0.911	0.001
Height (cm)	182.83(8.44)	183.33(6.50)	0.358	0.563	0.035
Weight (kg)	85.15(12.21)	80.50(14.58)	0.650	0.439	0.061
RWT	0.35(0.02)	0.36(0.01)	0.536	0.481	0.688
LVM	230.99(45.04)	137.16(19.17)	22.038	**0.001**	0.999
LVMI	111.33(16.41)	67.65(5.81)	37.736	**0.000**	0.791

RWT – Relative Wall Thickness; LVM – Left Ventricular Mass; LVMI – Left Ventricular Mass Index

## A9 Molecular hydrogen in sport environment

### Javorac Dejan, Valdemar Štajer, Darinka Korovljev, Slavko Molnar, Nebojša Maksimović and Sergej Ostojić

#### Faculty of sport and physical education, University of Novi Sad, Novi Sad, Serbia

##### **Correspondence:** Javorac Dejan (javorac.dejan@gmail.com)

**Background**

Molecular hydrogen (H2) emerged as a novel therapeutic agent, with antioxidant, anti-inflammatory and anti-apoptotic effects demonstrated in plethora of animal disease models and human studies. Molecular hydrogen is very rare in the Earth's atmosphere (1 ppm/vol) due to its lightness. It is one of the basic components of water and all organic matter as a result of its ability to form a covalent bond with most non-metallic elements [1]. Molecular hydrogen belongs to the category of innovative and attractive agents, which is proven by positive effects in various medical conditions, including acute and chronic diseases and injuries of the locomotor system. In particular, H2 may be an effective and specific treatment for exercise-induced oxidative stress, with a potential for the improvement of exercise performance [2,3]. H2 is the smallest molecule and can therefore easily penetrate the cell membrane and disperse rapidly in organelles. It is believed that H2 can selectively reduce hydroxyl radicals and peroxynitrite and does not affect physiologically reactive species. H2 can be ingested through several routes of administration, such as inhalation, oral administration of H2 water and H2 bathing.

**Methods**

The research on available literature was conducted on PubMed (Medline) database. All studies included provide evidence regarding the effects of H2 intake on changes in physiological and biochemical parameters focusing on human study in this research area.

**Results**

Following the literature review and after reading the abstract or full text, 6 studies were included in the review (Table 1). The evidence shows that there is a justified presumption that molecular hydrogen can be considered as an effective performance-enhancing agent. H2 improves sports performance, while on the other side it can reduce fatigue. Several human clinical studies have shown a promising therapeutic effect of hydrogen, applied in sports medicine with a focus on H2 as a new powerful agent for improving sports achievements [2,4]. For example, a Japanese group [5] concluded that daily intake of hydrogen-enriched water in the amount of 1.5 L (0.92-1.02 mM hydrogen) significantly reduces the level of lactate in blood and alleviates the decline in muscle function caused by exercise in football players. Additionally, two weeks of hydrogen application maintain maximum strength during repeated sprints to exhaustion lasting 30 minutes in cyclists [6]. Also, molecular hydrogen has shown to be a useful agent in relieving fatigue after moderate exercise on a bicycle ergometer and increasing endurance by measuring VO_2max_ [7]. Inhalation of 4% hydrogen for 20 minutes per day resulted in an increased maximum running speed (up to 4.2%) compared to the inhalation of air. Hydrogen inhalation resulted in a significant decrease in serum insulin-like growth factor 1 (IGF-1) of 48.2 ng / mL during monitoring, while IGF-1 levels were increased by 59.3 ng / mL after placebo intervention [3]. Molecular hydrogen may be helpful in improving the speed of recovery in soft tissue injuries [2].

**Conclusion**

Recently, numerous studies have indicated that molecular hydrogen influences cell transduction signals and acts as an alkalizing agent, so it has the potential to expand its application in clinical medicine to other spheres. Molecular hydrogen can be an effective and specific treatment for oxidative stress caused by exercise as well as for sports injuries, with the potential to improve sports performance. Previous research has not indicated negative consequences of molecular hydrogen on health, since top athletes are exposed to an increased physical and mental effort. For this reason, it is very important that future research is directed towards the impact on athletes as well as physically active population. In addition to many useful properties of molecular hydrogen, as well as the absence of unwanted, this paper aims to determine the impact on sports population. Since H2 is a gas molecule and has several advantages, it is worthwhile to continue research towards the application of H2 in sports science. In the meantime, more human studies with different doses and methods of administration are required before any recommendation is given or standard treatment applied in sports environment.

**References**

1. Ostojic SM. Molecular hydrogen in sports medicine: new therapeutic perspectives. Int. Sport. Med. 2014;36(04),273-279.

2. Ostojic SM, Calleja-Gonzalez J, Vukomanovic B, Hoffman JR. Effectiveness of oral and topical hydrogen for sports-related soft tissue injuries. Postgrad. Med. 2014:126(5):188-196.

3. Javorac D, Stajer V, Ratgeber L, Betlehem J, Ostojic S. Short-term H2 inhalation improves running performance and torso strength in healthy adults. Biol. Sport 2019;36(4):333-339.

4. Drid P, Trivic T, Casals C, Trivic S, Stojanovic M, Ostojic SM. Is molecular hydrogen beneficial to enhance post-exercise recovery in female athletes? Sci. Sports 2016:207-213.

5. Aoki K, Nakao A, Adachi T, Matsui Y, Miyakawa S. Pilot study: Effects of drinking hydrogen-rich water on muscle fatigue caused by acute exercise in elite athletes. Med. Gas 2012;12(2):12.

6. Ponte AD, Giovanelli N, Nigris D, Lazzer S. Effects of hydrogen rich water on prolonged intermittent exercise. J. Sports Med. Phys. Fitness 2018;58(5):612-621.

7. Mikami T, Tano K, Lee H, Lee H, Park J, Ohta F, et al. Drinking hydrogen water enhances endurance and relieves psychometric fatigue: a randomized, double-blind, placebo-controlled study. Can. J. Physiol. Pharmacol. 2019:97(9):857-862.

**Table 1 (abstract A9).** Study characteristics

Study	N	Study design	Intake protocols	Outcome
Ostojic et al. [2]	36	RT, SB	4 tablets x 3 oral (2g per day) 6 times per day for 20 min topical 14 days	Plasma viscosity ↓ (ROM) recovery ↑ CRP → Interleukin 6 → Pain scores →
Drid et al. [4]	8	RT, DB, PC, CO	300 mL of HRW 30 min before exercise 4 days	pH ↑ Bicarbonate (mmol/L) ↔ Lactate (mmol/L) ↓ Heart rate (beats per min) ↔
Aoki et al. [5]	10	RT, DB, PC, CO	500mL × 3 before exercise 7 days	d-ROMs, → BAP → CK → Lactate ↓ Peak torque ↑
Ponte et al. [6]	8	RT, PC, SB, CO	2 L per day (pH9.8, free hydrogen content 450 ppb) 14 days	PPO ↑ pH → Lactate → HCO_3_ →
Mikami et al. [7]	159	RT, DB, PC	500mL 0,8mh/L (0,8ppm) 1,0 mg/L (1.0ppm)	VO2max ↑ VAS ↓
Javorac et al. [3]	20	RT, DB, PC, CO	20 min inhalation 7 days	CRP ↓ FER ↓ ESR → IGF-1 ↓ MVIS ↑ MRS ↑ VO2max →

**Legend**: RT: randomized trial; DB: double-blinded; PC: placebo-controlled; CO: cross-over; SB: single-blinded; N: participants; HRW: Hydrogen-rich water; d-ROMs: diacron reactive oxygen metabolites; BAP: biological antioxidant potential; CK: creatine kinase; ESR: Erythrocyte sedimentation rate; CRP: C-reactive protein; IGF-1: Insulin-like growth factor 1; MVIS: Maximal voluntary isometric strength; VO2max: Maximal oxygen uptake; VAS: Visual analogue scale; FER: feritin; MRS: maximal running speed; PPO: Peak power output; (ROM) range of motion; ↑- increased; ↓: decrease; ↔ no change

## A10 Principles of acute body mass reduction in female sambo athletes

### Nikola Todorović^1^, Marijana Ranisavljev^1^, Borislav Tapavički^2^, Andrea Zubnar^2^, Jovan Kuzmanović^1^, Valdemar Štajer^1^

#### ^1^Faculty of Sport and Physical Education, University of Novi Sad, Novi Sad, Serbia; ^2^Faculty of Medicine, Department of Physiology, University of Novi Sad, Novi Sad, Serbia

##### **Correspondence:** Nikola Todorović (nikolatodorovic1708@gmail.com)

**Background**

SAMBO is a combat sport developed and used by the Soviet Red Army in the early 1920s to improve their combat abilities and physical fitness. The acronym SAMBO stands for "САМозащита Без Оружия", a Russian phrase that means "self-defense without weapons". Recently SAMBO received attention and recognition from the International Olympic Committee (IOC) and made the first steps toward inclusion into Olympic games. Weight categories have been used for centuries to equalize competition differences in several sports, especially in sports where physical advantages could influence the event's outcome. Earlier studies revealed that nearly 90% of Judo athletes involve rapid weight loss (RWL) prior to competition [1]. The same practices were noted among SAMBO competitors [2,3]. Further, concerning RWL strategies and athlete's health, RWL represents an important topic for discussion. Therefore, this cross-sectional study aims to compare senior and junior athletes regarding the influence and strategies adopted for RWL before competition.

**Methods**

The sample for this study were 47 females Sambo athletes (23.02±5.14 y/age, 1.63±0.8 m, 62.7±10.43 kg) from 12 countries, who competed in World Sambo Championship held in Novi Sad. They were further divided into two subgroups, seniors (27.3±4 y/age, 1.61±0.09 m, 61.8±8.87 kg) and juniors (18.7±0.8 y/age, 1.66±0.07 m, 63.7±12.1 kg). To determine RWL methods among sambo female athletes, we adopted the RWL questionnaire developed by Artioli et al. [4]. The questionnaire consists of questions concerning their RWL practices, including the methods used and the influence they receive during RWL protocols. For determining, the differences in RWL techniques and influences, we conducted chi-square tests and calculated frequencies. All the statistical analysis were conducted using SPSS statistical software (ver. 24.0) with significant levels set at p ≤ 0.05.

**Results**

The 88.7 % (n= 47) of female sambo athletes declared that they intentionally cut their weight before the competition. It was reported that they cut approximately 3.3 kg starting on average around 11 days before the competition. There were no statistical differences between groups concerning RWL methods. Athletes reported that gradual dieting (83 %), followed by sauna (72.9 %), fluid restriction (72.4 %), and skipping meals (70.4 %) were the most used RWL methods (calculated as the sum of the answers “always” and “sometimes”). The most significant influence in RWL procedures on athletes had coaches (67.4 %), while the less influential were physicians (12.7%) and dietitians (19.1 %) (calculated as the sum of the answers “somehow influential” and “very influential”) (see detailed in Table 1.). The only statistically significant difference between subgroups was noted in the influence of fellow sambist (25 % vs 21.7%), and it seems that overall experience sambist share their experience concerning strategies for RWL.

**Conclusion**

In this study, we evaluated RWL techniques among elite female SAMBO athletes. The majority of the athletes being assessed (88.7 %) cuts their weight prior to competitions. We did not find any differences between juniors and seniors. Concerning the influence, the most influential person in both categorize was the coach. Further, more experienced athletes more often seek advice from other fellow sambist. It also concerns that only a handful of athletes consult dietitians or physicians. In a mission to develop and improve SAMBO as a sport, further direction must consider the education of coaches and athletes concerning RWL techniques.

**Reference**

1. Artioli GG, Gualano B, Franchini E, Scagliusi FB, Takesian M, Fuchs M, Lancha Jr AH. Prevalence, magnitude, and methods of rapid weight loss among judo competitors. Med. Sci. Sports Exerc. 2010;42(3):436-442.

2. Drid P, Figlioli F, Lakicevic N, Gentile A, Stajer V, Raskovic B, Vojvodic N, Roklicer R, Trivic T, Tabakov S, Eliseev S. Patterns of rapid weight loss in elite sambo athletes. BMC Sports Sci. Med. Rehabil. 2021;13(1):1-7.

3. Figlioli F, Bianco A, Thomas E, Stajer V, Korovljev D, Trivic T, Maksimovic N, Drid P. Rapid Weight Loss Habits before a Competition in Sambo Athletes. Nutrients 2021;13(4):1063.

4. Artioli GG, Scagliusi F, Kashiwagura D, Franchini E, Gualano B, Junior AL. Development, validity and reliability of a questionnaire designed to evaluate rapid weight loss patterns in judo players. Scand. J. Med. Sci. Sports. 2010;20(1):e177-87.

**Table 1 (abstract A10).** Frequency distribution of athlete’s influential persons during RWL

Source of influence	Group	Not influential	Little influential	Unsure	Somehow influential	Very influential	χ2	***p***
Fellow sambist	Seniors	13 (54.2 %)	0 (0%)	5 (20.8%)	4 (16.7%)	2 (8.3%)	11.5	0.02*
	Juniors	9 (39.1%)	8 (34.8%)	1 (4.3%)	3 (13%)	2 (8.7%)		
Physician	Seniors	12 (50%)	8 (33.3%)	0 (0%)	1 (4.2%)	3 (12.5%)	6.03	0.3
	Juniors	16 (69.6%)	3 (13%)	1 (4.3%)	0 (0%)	2 (8.7%)		
Coach	Seniors	3 (12.5%)	2 (8.3%)	0 (0%)	11 (45.8%)	7 (29.2%)	5.02	0.29
	Juniors	5 (21.7%)	3 (13%)	2 (8.7%)	5 (21.7%)	8 (34.8%)		
Dietitian	Seniors	15 (62.5%)	2 (8.3%)	2 (8.3%)	2 (8.3%)	3 (12.5%)	3.31	0.51
	Juniors	17 (73.9%)	0 (0%)	2 (8.7%)	3 (13%)	1 (4.3%)		

Legend: 2-chi-square test; p-probability; *-significant

## A11 The role of physical activity in the quality of life and sedentary behaviour of children and adolescents

### Darinka Korovljev^1^, Nikola Todorovic^1^, Valdemar Stajer^1^, Nebojsa Maksimovic^1^, Sergej Ostojic^1,2^

#### ^1^Applied Bioenergetics Lab, Faculty of Sport and Physical Education, University of Novi Sad, Novi Sad, Serbia; ^2^Faculty of Health Sciences, University of Pecs, Pecs, Hungary

##### **Correspondence:** Darinka Korovljev (korovljev.darinka@gmail.com)

**Background**

Numerous studies have shown that more physical activity and less time spent in a sedentary lifestyle are associated with the overall better health-related quality of life (HRQOL) in children and adolescents. To date, very few studies have been conducted that synthesize the link between physical activity, sedentary behaviour, and quality of life-related to health in children and adolescents. The objective of this study was to review the associations between physical activity, sedentary behaviours, and HRQOL in children and adolescents aged < 19 in a general and clinical population.

**Methods**

The research was conducted by reviewing all available relevant scientific articles within the PubMed database. Initially, we found 108 articles, including clinical trials and randomized clinical trials in English for the period 2016 to 2021. Further analyses identified 6 studies that evaluated the connection between physical activity (PA), sedentary behaviour (SB), and health-related quality of life (HRQOL) in children and adolescents aged < 19 years in a general and clinical population. After a more detailed analysis of initial studies, the research was reduced to three studies, and three studies were excluded from further analysis: one describing the research protocol (e.g., no research data), one that did not meet the criteria for HRQOL, and another concerning the cost analysis. PUBMED search employed the following keywords: "health-related quality of life," "sedentary behaviour," "physical activity," "children," and "adolescent."

**Results**

The results of our analysis are depicted in Table 1. The studies involved a total sample of 62 children and adolescents aged < 19 years, and HRQOL was monitored after different types of health interventions in children with chronic conditions, including cerebral palsy, cystic fibrosis, and heart transplantation.

Moola et al. [1] designed a study to assess the feasibility of theoretically informed 8-week counselling intervention through parents in increasing habitual physical activity and quality of life of children and adolescents with CF (n=13, age 8-18 y). The intervention was found to be feasible and acceptable with good recruitment, retention, adherence, and acceptability. There have also been positive trends in terms of increased PA, reduced sedentary time, and improvements in most dimensions of HRQOL comparing the period before the intervention with the period after the intervention, suggesting that counselling is feasible for the CF community. Another interesting study [2] evaluated motivational and behaviour change therapy intervention to increase PA and HRQOL through meaningful participation in children with cerebral palsy in an 8-week exercise program (n=18 men; aged 8-12). The results of the study showed that there were no statistically significant effects after the intervention in the HPA indicators, but there was an increase in the MPA values. A recent study by Chen et al. [3] recruited children and adolescents aged 8-19, who had a heart transplant a year ago, who had confirmation from their cardiologist, and a caregiver or adult at home during exercise, especially for persons under 14 years of age. The intervention consisted of daily application of the diet and exercises three times during the week via video conference. This study confirmed that this type of intervention through diet and exercise was applicable, which had a positive effect on improving VO2max and physical functioning, a surrogate marker of HRQOL.

**Conclusions**

According to studies included in our analysis, health interventions that promote active lifestyles for children and adolescents may contribute to improve health-related quality of life. One of the limiting factors is certainly the small number of sample participants. It can be concluded that health-related quality of life in children and adolescents, whether healthy or with chronic conditions, can be improved with the help of designed long-term health interventions and studies involving the use of dosed habitual and moderate physical activity, as well as the adoption of healthy diets and the reduction of SB. Future research is certainly needed to expand studies and longitudinal links between physical activity, sedentary behaviour, and health-related quality of life in children and adolescents.

**References**

1. Moola FJ, Garcia E, Huynh E, Henry L, Penfound S, Consunji-Araneta R, Faulkner GE. Physical activity counseling for children with cystic fibrosis. Respir. Care 2017;62(11):1466-1473.

2. Reedman SE, Boyd RN, Elliott C, Sakzewski L. ParticiPAte CP: a protocol of a randomised waitlist controlled trial of a motivational and behaviour change therapy intervention to increase physical activity through meaningful participation in children with cerebral palsy. BMJ Open 2017;7(8):677-686.

3. Chen AC, Ramirez FD, Rosenthal DN, Couch SC, Berry S, Stauffer KJ, Brabander J, McDonald N, Lee D, Barkoff L, Nourse ES, Kazmuha J, Wang CJ, Olson I, Tierney ESS. Healthy hearts via live videoconferencing: an exercise and diet intervention in pediatric heart transplant recipients. J. Am. Heart Assoc. 2020;9(3):1-17.

**Table 1 (abstract A11).** Studies evaluating the effects of health interventions on HRQOL in children and adolescents

References	Subjects	Health Interventions	Main outcomes
[1]	(*n* = 13) Age = 8-18 yrs	8-week counselling program 12 week PA intervention moderate to vigorous sessions 2 x week 90 min; 12 week follow–up HRQOL	Feasibility counselling program ↑ PA min/day ↓ SB min/day ↑ HRQOL: Physical; School and Emotional domain
[2]	(*n* = 36) Age = 8-12 yrs	8-week exercise program ParticiPAte	↑ Physical activity min/day (MPA) ↔ CP QoL
[3]	(*n* = 13) Age = 8-19 yrs	12-16 week diet and exercise intervention to improve cardiovascular health via video conferencing 3x per week	↑ %VO2 max ↑Functional movement score

*Abbreviations*: HRQOL – Health-related quality of life; PA – physical activity, SB - sedentary behaviour; CP – cerebral palsy QoL – Quality of Life; MPA – moderate physical activity; ↑– significant increase; ↓– significant decrease; ↔ no change; VO2 max – maximal oxygen consumption

## A12 Bioethics and Sport – Meeting Points

### Sandra Radenović (sandra.radenovic@fsfv.bg.ac.rs)

#### Faculty of Sport and Physical Education, University of Belgrade, Belgrade, Serbia

**Background**

It is considered that sport is a multidimensional and complex phenomenon whose backbone is competition, and besides the competition as an essential element, the field of sport includes social, historical, psychological, economic, political, pedagogical, medical, ethical, philosophical, religious, environmental, cultural, legal, technical-technological, aesthetic and numerous other aspects [1]. All the mentioned aspects are the subject of the study of humanities, and in this lecture, we will focus on the bioethical and sociological aspects of professional sports. Firstly we will explain the importance of the bioethics of sport as a philosophical discipline and a wider interdisciplinary field. In a narrow sense, bioethics of sport focuses on critical consideration of doping, bribery, and corruption in professional sports, but also on the problem of medical ethical principles, for example, the principle of informed consent addressed to an injured athlete by a sports medical physician, as well as ethical dilemmas in choosing between quality medical recovery and rapid recovery of the athlete in order to return to the field as soon as possible, etc. In a broader sense, the bioethics of sport deals with all those ethical aspects of the sport, both in sports medicine and in humanities, such as the sociology of sport. Hence, the bioethics of sport considers ethical aspects of the context and social conditioning of sport, the impact of society on sport and vice versa – the impact of sport on society, ethical aspects of numerous interactions between all elements of a sports events - players, mediators, fans and sports audience, ethical aspects of sports reporting by sports journalists as the most important mediators of every sports event, ethical aspects of the connection between national identity and sports, the problem of racism in sports, the representation of women in sports and gender inequalities in sports, etc. [2,3]. As sociology of sport is a special sociological discipline that studies sport as a complex socio-cultural and historical phenomenon and the relationship between sport and society [4], it is clear that the bioethics of sport in its broadest sense interferes with the sociology of sport regarding the topics related to professional sports and the complex relationship between sports and society. The unstoppable development of medicine and technical-technological development of the last few decades has brought with itself certain new problems and ethical dilemmas, both at the level of global society and in the field of professional sports. For example, strong development in medicine and technology has made something that once was unthinkable – now it is the everyday life of people with disabilities who are allowed to compete even though they do not have a limb or limbs. We consider the phenomenon of human cyborgization, which means the formation of a cyborg, a combination of "living" and "non-living," i.e., a combination of man as a living organism and an artificial work, in this case, artificial lower extremities. The example of cyborgization clearly indicates the changes in professional sports are caused by huge changes, more precisely – by the progress of medicine and technology in the modern age. At the same time, cyborgization raises a number of ethical questions, such as how to achieve fairness in competition between "natural" and "cyborgized" athletes, whether "natural" and "cyborgized" athletes may compete at all, or whether cyborgization creates artificial, cyborgized winners, etc. There are no final answers to these and similar questions, but certainly, that huge and unstoppable changes in technology and medicine directly affect changes in sports, opening numerous bioethical and sociological questions, i.e., ethical dilemmas that deserve serious analysis [3]. Finally, let us mention the problem of so-called technological doping, which refers not only to the improvement of sports equipment, but also to, for example, the quality of the running track, and which may be one of the reasons for numerous athletic records at the recent Olympic Games in Tokyo.

**Conclusions**

Hence, it is considered that the mentioned problems and ethical dilemmas which are present in contemporary professional sports open the possibility of establishing ethics committees in sports as the bioethical bodies which consider the gap between law and ethics [1]. Future ethics committees in sport may be specific bioethical bodies that will seriously consider such and similar mentioned examples and provide valid, acceptable solutions that will not violate the ethical principles of the sport. This lecture points to the fact that the mentioned examples reflect the complexity of professional sports, which is increasingly conditioned by the unstoppable development of technology and medicine. That is why the given examples and the newly arisen ethical dilemmas and current problems are 'the burning' meeting points between bioethics as a broad interdisciplinary field and professional sports as a complex, multidimensional socio-cultural and historical phenomenon.

**References**

1. Radenović S, Mijatov N. Ethical committees and professional sports – ‘bioethicalization’ of sports as a need. Sport Soc. 2020; doi: 10.1080/17430437.2020.1822820.

2. Škerbić MM, Radenović S. Bioetika sporta: prisutnost bioetičkih tema na području filozofije i etike sporta u Hrvatskoj i Srbiji [Bioethics of Sport: The Presence of Bioethical Topics in the Field of Philosophy and Ethics of Sport in Croatia and Serbia]. JAHR 2018; 9(2): 159-184. In Croatian.

3. Radenović S. Sport i društvo – Sociologija sa sociologijom sporta [Sport and Society – Sociology with Sociology of Sport]. Belgrade: Faculty of Sport and Physical Education, University of Belgrade. In Serbian.

4. Milovanović I. Uvod u sociologiju i sociologiju sporta [Introduction to Sociology and Sociology of Sport]. Novi Sad: Faculty of Sport and Physical Education, University of Novi Sad. In Serbian.

## A13 Sociology of sport – is it going through innovation or fragmentation?

### Ivana M. Milovanović (i.a.milovanovic@gmail.com)

#### Faculty of Sport and Physical Education, University of Novi Sad, Novi Sad, Serbia

**Background**

In this paper, the author presented the development of the sociology of sport as a relatively new scientific discipline. In recent years, the sociology of sport has faced an ambivalent process of apparent innovation (reflected in the establishment and development of related sub-disciplines), which represents a fragmentation of sociology of sport and may lead to a crisis of subject identity for the discipline as a whole.

**Methods**

Referring to secondary literature on the sociology of sport [1-3], the author describes the process of the "separation" of sport as social activity from play/game and leisure. In the second half of the XX century, this process was dependent on market relations in society as a whole, especially the rapid development of professional/commercial sport, cable television, the separation of sport TV channels, and the subsequent development of the sponsorship and advertising commercials concept [2]. Through the disciplinary (mainly empirical) development of the sociology of sport, the sport itself gains a clearer cognitive legitimacy, first in sociology and then in other social sciences and humanities.

**Results**

The aforementioned development of the sociology of sport, especially in the late XX century and the first decade of the XXI century, indicates contradictory tendencies specific to the sociology of sport itself. Namely, researching topics such as commercialization of sport, raising some sport and sports events to the level of spectacle, the insistence on results, the breaking records, the imperative to win, the development of idolatry in sport, the study of fans behaviour (primarily destructive), the social influence of the mass media through sports news, led to an increasingly evident fragmentation of the sociological theoretical approach to the sport itself as social phenomenon [4]. This is most evident in the establishment and development of related sociological sub-disciplines (such as sociology of sports training, sociology of football, *sociology* of Christian Ronaldo), whose theoretical approach has no clear connection with a more general sociological approach. Such tendency suggests that scientific differentiation into disciplines could be the evidence of their growth and development, but not necessarily progress because, during the process of fragmentation, the need for the universalization of knowledge is necessarily lost. This further points to the application of the new positivist logic in science, which had led to numerous scientific achievements in recent decades, strongly reflected in the specialization of knowledge. On the other hand, the "innovative" approach itself is dangerous for social sciences, which strive for universal knowledge, because the uncontrolled fragmentation of science into more and more sub-disciplines leads to disciplinary "anarchy." In this way, researchers are left with partial data and results without the possibility of generalization as one of the core principles of scientific knowledge. The sociology of sport itself remains at the "crossroads" between the need to develop and the risk of remaining an incomplete sociological discipline, especially since sociology of sport, despite its decades of existence, has not yet substantially demonstrated all social aspects of the sport. The reason for the "vagueness" is to be found in rapid (mainly empirical) development from the 1960s to the 1990s in the USA [5]. The discipline has developed but without a clear theoretical and methodological connection to general sociology, unlike other sociological disciplines. Moreover, sport is an essential part of the culture from which it originates [6]. It has evolved and changed along with the cultural values of each society, which further hindered the overall development of the discipline.

**Conclusions**

The development of the sociology of sport has been twofold for several decades; as a part of general sociology/its parent science and within sport science. Therefore, one should not be surprised by the directions of its disciplinary growth and development, which puts general sociological elaborations of the social dimension of sport into the background and gives priority to certain" pragmatic" and actual research topics, which are mere fragments of social life. The result is that the sociology of sport does not behave "on an equal footing" with the already established sociological disciplines but continues to "develop" independently through new related sub-disciplines, running the risk of losing its professional identity over time. That would cause a further need for reconsideration of the theoretical basis of this important sociological discipline that studies sport in order to understand the whole socio-historical context. This is important due to the fact that the sport is a significant social activity in contemporary society, whose further development should be sociologically elaborated. Otherwise, the research results would be a mere sum of fragments without showing their core and relationship to the broader social context.

**Acknowledgments**

Big thanks to my colleagues Assoc. Prof. Sandra S. Radenović and ass. Prof. Saša Pišot for joint contemplation and exchange of ideas, which are partly incorporated in this abstract.

**References**

1. Coakley J. Sport in Society: Issues and Controversies. New York: McGraw-Hill; 2007.

2. Delaney T, Madigan T. The Sociology of Sport: An Introduction, Second edition, North Carolina; McFarland & Company, Inc., Publishers, Jefferson; 2014.

3. Gulianotti R. Sport: Kritička sociologija [Sport: Critical Sociology], Clio, Belgrade; 2008.

4. Milovanović I, Radenović S. Contemporary tendencies in the sociology of sport: From constitution to "fragmented" discipline. Sociology 2020;62(2):37-254.

5. Milovanović I. Uvod u sociologiju i sociologiju sporta [Introduction to Sociology and Sociology of Sport], Novi Sad: Faculty of Sport and Physical Education. 2017.

6. Radenović S. Sociology of sport and/or sociology of physical culture? – Certain considerations. Sociološka luča 2015;IX(1):42–55.

## A14 How far will we go? The influence of high technology and innovation in sports on some social categories

### Saša Pišot (sasa.pisot@zrs-kp.si)

#### Science and Research Centre, Koper, Slovenia

**Background**

Under the impression by the completed Tokyo 2020 Olympics, some extreme performance on the track, along with another 26 records broken, have led to speculation about the impact of new technology in sport. Coupled with the news that two sprinters have been banned from the Olympics after failed doping tests, new questions are being raised: How far will we go? And will we continue to break records using or abusing technology and innovation in sport? Despite constant warnings that we are close to the limit of human performance, records will keep be broken for many reasons, from advances in technology to changes in rules and society.

**Methods**

Research into the latest evidence, data from the Tokyo Olympics, and secondary literature in the field of sociology of sport were used to analyze, from a sociological perspective [1], the factors that help us explain the reasons for the persistence of the world record rate. In addition, some sociological categories such as inequality, exclusion, deviance, and health problems were analyzed in relation to the position and impact of high technology and innovation in sport.

**Results**

***Factors of record-breaking world phenomenon.*** Studies in 2005 and 2007 [2] analyzed world records over time (running and swimming), found they followed an S-shaped curve. Due to scientific improvements in training, coaching, and nutrition, as well as social factors and the globalization of sports, records in all sports made a huge jump in the middle of the 20^th^ century. Since then, the rate of record-breaking has slowed but still exists. Olympians in Tokyo set 26 new world records; in Rio 2016, broke 27, in London 2012, 32, and in Beijing 2008, 34 [2,3]. Factors to determine how many world records are broken are the increased pool of the athletes due to i) geographical expansion (more countries gain access to Olympic competitions); ii) new rules of professionalism (after decades of strict amateur code, by 1992, the Olympics allowed professional athletes to compete, also from poorer countries), iii) longer athletes careers (financial accessibility combined with improvements in sports medicine), iv) technological advances and sports innovation (artificial snow and ice allow year-round training; optimal depth and temperature in the swimming pools, the shock-absorbing rubber technology of the Tokyo Olympic track should give athletes a performance advantage of about 1-2% [2]. When it comes to winning and a chance to break world records, any technological trick is used to gain milliseconds, every little advantage counts and leads to a "technological arms race." It is not just about designing faster equipment and suits but also about improving the physical abilities of athletes through the use of the latest medical and biological research. In a race to be the best, the abuse of biomedical research by athletes and their coaches to gain an unfair advantage; for decades, professional sport has been tainted by doping—the use of illegal substances.

***The influence of high technology and innovation in sports on some social categories*** **.**

*Deviant behaviour and health issue.* Athletes today run, jump or swim not only for glory but also for money – after all, a gold medal or a world record is the ticket to lucrative advertising contracts. However, to achieve that glory, athletes are willing to train hard, giving up ordinary life, push and test their body limits and take the shortcuts through doping. Some of them use illegal substances such as erythropoietin (EPO), steroids or growth hormones and others, that were originally developed to treat human diseases but have also been used to boost the performance of healthy athletes [4]. Besides the scientific evidence of harmful effects of doping on health also the risk of competing when injured and risky training regimes are definitely issues that should not be ignored when considering the health of athletes.

*Exclusion in sport*. In addition to the traditional factors of exclusion (gender and race), we focus on disability in sport. Despite the fact that the Paralympic Games movement enable disable athletes to be included in sport, but still, in the world of innovation, the question of whether or not prosthetic limbs give disabled athletes an unfair advantage over competitors with biological limbs arises daily [5] as was argued in the Oscar Pistorius’ case at London Olympic, 2012 [6].

*Inequity and equal opportunities in sport.* Despite geographical expansion and globalization of sport, in terms of the number of Olympic athletes per capita, there are still many (third world) countries struggling under their weight of sport participation: India (125 athlete/ 1.4 billion people), Indonesia (28 athletes/ 274 million) and 26 million North Koreans who do not have access to the games [2]. These facts raise the question of inequity. Also, the sport’s governing bodies should always look at technical innovation and decide that any new gear has to be “reasonably available” to everyone. In practice is not always so fair to all (e.g., swimmers in Beijing wearing a Speedo LZR Racer suit (by NASA) won 94% of all races, 98% of all medals, and broke 23 world records, the suit was banned from competition). Sport today both reinforces forms of social inequality [7] and provides a (financial) resource of hope for some people.

**Conclusions**

The description of the social categories affected by technological innovation in sport draws attention to the need for multidimensional research of consequences, in particular to those that are harmful to the athlete's well-being. Being aware that greed for money will not stop technological development, it is necessary to educate athletes, sports, and the public sphere about what lies behind medals and records. On the opposite, we will assume the prevailing mind-set of celebrating technological advances and surgeries on bodies to do maximum performances [4], forgetting the nature of sport and Olympic games – celebrating the human will overcoming obstacles.

**Acknowledgments**

Thanks to my colleague, prof. Ivana Milovanović for productive discussions and exchange of ideas that are successfully implemented in joint work.

**References**

1. Houlihan B, Malcolm, D (Eds.). Sport and society: a student introduction. Sage;2015.

2. Inverse [https://www.inverse.com/science/olympic-world-records-broken-2021-science], accessed date 10 July 2021.

3. Sportstar [https://sportstar.thehindu.com/olympics/tokyo-olympics/olympics-world-record-tokyo-games-2020-dressel-warholm/article35829713.ece], accessed date 10 August 2021.

4. Schneider AJ, Friedmann T. Gene doping in sports: the science and ethics of genetically modified athletes. Adv. Genet. 2006;51:1–110.

5. Engineering sport [https://engineeringsport.co.uk/2011/10/07/the-trouble-with-oscar/] accessed date 10 July 2011.

6. Amputee store [https://amputeestore.com/blogs/amputee-life/do-blade-prostheses-give-amputee-runners-an-advantage/], accessed date 9 September 2019.

7. Jarvie G. Sport, social division and social inequality. Sport Sci. Rev. 2011;20(1-2):95-109.

## A15 Psychological distress in elite sambo athletes

### Tatjana Tubić, Patrik Drid

#### Faculty of Sport and Physical Education, University of Novi Sad, Novi Sad, Serbia

##### **Correspondence:** Tatjana Tubić (tttubic@gmail.com)

**Background**

Previous research suggests that as much as playing sports has a beneficial effect on mental health, playing sports, especially at the elite level, is also a mental health challenge. Main objective of this study is determining the variations in the prevalence of psychological distress among athletes, elite sambo and recreational.

**Methods**

Sample consisted of 245 athletes, of both genders (127 males and 118 females); out of the total sample, 105 are elite sambo athletes who participated in European Sambo Championship in Cyprus (May, 2021) and 140 are recreational athletes. Those participations completed The Depression Anxiety Stress Scales-21 (DASS-21) [1]; the DASS was shown to possess satisfactory psychometric properties, Cronbach`s Alpha in a range of 0.775 to 0.897, while indicators of distribution normality indicate that most athletes scored low scores in examined subscales of depression, anxiety and stress, and in general distress, with a large deviation from normality.

**Results**

Results obtained by appropriate non-parametric measures indicate that elite sambo athletes achieve statistically significant lower scores in all examined variables of psychological distress in comparison with recreational athletes. Although, when observing total athletes sample there is no gender differences in psychological distress, within female elite sambo athletes achieve statistically significant lower scores in all examined variables than recreational ones. Females who practice recreational activities were distinguished as vulnerable subsample in terms of psychological distress.

**Conclusions**

Obtained variations in prevalence of psychological distress in examined athletes subsamples present valid guidelines for the future investigations growing emphasis to provide specific-sport and gender-dependent support for mental health issues.

**Reference**

1. Lovibond SH, Lovibond PF. The structure of negative emotional states: Comparison of the Depression Anxiety Stress Scales (DASS) with the Beck Depression and Anxiety Inventories. Behav. Res. Ther. 1995:33:335-343.

## A16 The importance of the promotion of sports entrepreneurship: an analysis of the entrepreneurial intentions of sports science students at University of Novi Sad

### Radenko Matić (radenkomatic@uns.ac.rs)

#### Faculty of Sport and Physical Education, University of Novi Sad, Novi Sad, Serbia

**Background**

The difficult position of students after graduation of studies caused that unemployment is a problem worldwide. One essential factor in decreasing the youth unemployment rate is entrepreneurship as an integral part of every professional industry [1]. So influential status of entrepreneurship renewed the scientific focus of many researchers on entrepreneurship from different perspectives, including the sports sciences. Further, predicting factors with impact on the entrepreneurial intentions (EI) of sports science students (SSS) as potential future entrepreneurs can be found as a topic in many types of research [2]. These studies imply that promoting sports entrepreneurship (SE) during the studying encourages future entrepreneurs to create successful business projects. Therefore, the proposed theoretical model in this study was based on the Theory of Planned Behaviour as one of the most accepted theories in predicting human behaviour. Thus, the following five hypotheses are presented: 1) Attitude toward behaviour (ATB) has a positive direct relationship with EI, 2) Perceived Behavioural Control (PBC) has a positive direct relationship with EI, 3) Subjective Norm (SN) has a positive direct relationship with EI, 4) ATB, SN, and PBC mediate the relationship between Entrepreneurial skills (ES) and EI, 5) ATB, SN, and PBC mediate the relationship between Country Culture (CC) with EI. This study aimed to test the influence of ES, CC, and characteristics of planned behaviour on the EI of SSS to become future sports entrepreneurs.

**Methods**

The sample of respondents included 96 males and 31 females of SSS (N=127) from the final year at the Faculty of Sport and Physical Education, University of Novi Sad, Serbia. All students were informed about sports management. The online survey was realized during the academic 2020/2021 year. This questionnaire consisted of the following scales: *External dimensions:* ES – 6 items (different skills related to entrepreneurship), which were extracted from Liñán [3] and CC – 5 items (e.g., The entrepreneur's role in the economy is not sufficiently recognized); *Mediators:* ATB – 5 items (e.g., Being an entrepreneur implies more advantages than disadvantages); SN – 3 items (e.g., Your Friends, family, etc.), PBC – 6 items (e.g., I can manage the process of developing a new firm). These scales were extracted from the Entrepreneurial Intention Questionnaire by Liñán and Chen [4]; *Dependent variable:* EI – 6 items (e.g., I am ready to do anything to be an entrepreneur). All scales were measured with Likert-type scales, ranked from 1 (strongly disagree) to 7 (strongly agree). Statistical analysis was based on testing the relations and paths among latent dimensions in the theoretical model. Data analysis considered running the Confirmatory factorial analysis and Structural equation modelling using the Smart PLS version 3.3.2. Additionally, statistical indicators of the proposed models' goodness-of-fit indices were ran using SPSS, AMOS, version 24.0.

**Results**

The reliability and validity of the measurement model were on an acceptable level (>.73), such as composite reliabilities (CR greater than 0.82) and Average Variance Extracted (AVE - from 0.50 to 0.82). Also, all factor loadings satisfied the criteria with more than 0.4. The fit indices showed acceptable values (χ2 = 706.48, df =425, χ2/df = 1.66, CFI = 0.91, NNFI= 0.91, IFI = 0,91 and RMSEA = 0.05). Directs significant effects were evidenced between ATB (β = 0.64) and PBC (β=0.31) and EI, which supported accepting hypotheses 1 and 2. On another side, the direct effect of SN and EI was not confirmed, which contributed to Hypothesis 3 was rejected. Further, the analysis of the indirect effect of ES on EI revealed a statistically significant indirect relationship among dimensions (β = 0.39), which partially confirmed the H4 hypothesis (mediated by ATB and PBC). Lastly, hypothesis 5 was rejected because AT, SN, and PBC do not mediate between CC and EI.

**Conclusions**

This study is conceptualized to predict the crucial factor which determines the EI of SSS. From that perspective, findings revealed positive and direct effects of ATB on EI, which is found out in numerous studies [5,6]. A higher value of the path regression coefficient showed interest in SSS for future SE. Another statistically positive direct impact on EI was highlighted from PBC. The only dimension of SN has no significant effect on EI. In that context, these results are in line with Serrano et al. [7]. These authors explained the influence of gender and academic training on EI of pre-graduate (1st-4th course) and post-graduate of Physical Activity and Sport Science degree of Valencia. The results showed that ATB and the PBC were the predictors for both genders, while the SN has an important role only for men. From another aspect, it has revealed the role of SN [4]. If we compared the impact of internal and external dimensions on EI, it's evident that students based their EI more on internal factors (ATB and PBC) than external dimensions. University could improve the perceptions of these internal dimensions, which is proposed by Liñán [3]. Although the University of Novi Sad strives to be an entrepreneurial university, this effort is not enough to create a favourable entrepreneurial climate for SSS. Therefore, university education and policymakers should actively promote students' SE and contribute to ES and attitudes. Such strategy can decrease the youth unemployment rate.

**Funding**

The data used in this study were collected within the research project "The importance of the promotion of sports entrepreneurship: an analysis of the entrepreneurial intentions of sports science students" (register number: 142-451-2258/2021-01), which was conducted by the Faculty of Sport and Physical Education and financed by the Provincial Secretariat for Higher Education and Scientific research.

**References**

1. Ratten V. Sport-based entrepreneurship: Towards a new theory of entrepreneurship and sport management. Int. Entrepreneurship Manag. J. 2011;7(1):57-69.

2. González-Serrano MH, Calabuig Moreno F, Valantine I, Crespo Hervás J. How to detect potential sport intrapreneurs? Validation of the intrapreneurial intention scale with sport science students. J. Entrepreneurship Public Policy 2019;8(1):40-61.

3. Liñán F. Skill and value perceptions: how do they affect entrepreneurial intentions?. Int. Entrepreneurship Manag. J. 2008;4:257-272.

4. Liñán F, Jaén I. A proposed model for the culture's mode of influence on the entrepreneurial process, 62-83. In: M. Brännback AL. Carsrud (Eds.) *A research agenda for entrepreneurial cognition and intention*. Edward Elgar Publishing. 2018.

5. Munir H, Jianfeng C, Ramzan S. Personality traits and theory of planned behavior comparison of entrepreneurial intentions between an emerging economy and a developing country. Int. J. Entrepreneurial Behav. Res. 2019;25(3):554-580.

6. Naia A, Baptista R. Biscaia R, Januário C, Trigo V. Entrepreneurial intentions of Sport Sciences students And Theory of Planned Behavior. Mot. Rev. Educ. Fis. 2017;23(1):14-21.

7. Serrano MHG, Valantine I, Campos CP, Berenguer SA, Moreno FC, Hervas JJC. The influence of gender and academic training in the entrepreneurial intention of physical activity and sport sciences students. Intang. Cap. 2016;12(3):759-788.

## A17 The effectiveness of core stabilization on posture in adolescents young adults

### Tijana Šćepanović (tijanascepanovic021@gmail.com)

#### Faculty of Sport and Physical Education, University of Novi Sad, Novi Sad, Serbia

**Background**

Body posture is most often investigated at the age of children growing and developing. A low level of physical activity of children in early childhood, the use of modern technologies, and inadequate nutrition results in a risk for chronic metabolic and cardiovascular diseases. Children are becoming more obese, and they, therefore, have underdeveloped muscles, which represent a poor basis for proper body posture. Researchers have not often studied the posture of adolescents young adults of the age of 20 and more. However, we believe that it is possible to have a positive effect on posture at this age as well. In this study, the core treatment (CT) will be applied in order to strengthen the muscles of the trunk at the front and the backside of a body, resulting in a body balance and increased flexibility. The CT positively affects the entire posture [1]. The aim of the study was to determine the effects of a 6-week CT on the sagittal plane posture.

**Methods**

The sample was composed of 138 male students from the Faculty of Sport and Physical Education (University of Novi Sad, Serbia). All examinees were previously identified with postural changes visible from the sagittal plane, after which they were randomly divided into two groups ([experimental group (EG): n=73; age 20±0.5 years; height 1.80±0.05m; mass 76±9.4kg] [control group (CG): n=65; age 20±0.5 years; height 1.81±0.08m; mass 78.6±4.7kg]). Postural disorders were assessed using the Contemplas GmbH Templo photometric apparatus. The posture score is evaluated using a camera system and software analysis to determine the position of marked points in space according to the 2D (Fröner posture index (PI); Kyphose-inclination-lordosis (KIL scheme) and 3D analysis protocols. This method accurately measures the posture of body segments and asymmetrical posture. The assessors placed 16 reflective markers per examinee, after which they placed the examinees in a calibrated space and photographed them with three cameras (Basler acA645-100gm/gc). The CG received no intervention. The CT lasted 6 weeks and was performed three times a week. This was a total of 18 basic courses lasting 30 minutes each. The CT included exercises to increase the mobility and strength of the neck, shoulders, pelvis, and hips, and anti-flexion, anti-extension, and rotation exercises were used to stabilize the spine [2]. The intensity of those training courses was individual, but each one started at submaximal. In two weeks, the intensity was changing following the progress of each examinee. Data were analyzed by using SPSS (version 20.0, IBM Corp., Armonk, NY, USA). Mean and standard deviation for the pre-and post-test were calculated for each group. There was a normal distribution for both groups, p>0.05. Effects after CT have been demonstrated by Two-way repeated measures Anova.

**Results**

The results from repeated measures by Anova showed a significant group (EG vs. CG) × time (Pre to Post) interaction for 3D thoracic (F _(1,136)_ = 5.51, p = 0.02, Partial ƞ^2^ = 0.03), lumbar (F _(1,136)_ = 13.424, p = 0.00, Partial ƞ^2^ = 0.09), and KIL cervico-lumbar lead angle (F _(1,136)_ = 11.69, p = 0.00, Partial ƞ^2^ = 0.07). In addition to the significance mentioned above, the significant main effect of time was shown in KIL thoracic kyphosis index (F _(1,136)_ = 10.97, p = 0.00, Partial ƞ^2^ = 0.07). The research started with the aim of proving the effect of CT on positive posture changes in the sagittal plane. By analyzing the effects after the application of the CT, it can be concluded that there were positive changes in the spinal column, and mostly in the thoracic and lumbar segment. In the neck segment, posture changes are not statistically significant, which is logical because the treatment exercises focused on strengthening the muscles of the central part of the torso. Posture was analyzed through different 2D and 3D protocols. By 3D analysis protocol, the thoracic distance was drastically reduced in EG, from -2.52 to -1.65. In a study of adolescents up to 14 years of age, the average of the results is around -1,032 in healthy [3]. With age, in healthy subjects, the cervical spine decreases, and the thoracic spine increases [4]. This would mean that the trend with healthy people is to move the head forward by moving the head forward and increase the thoracic spine. Since we had a sample of respondents with pre-existing sagittal problems, any reduction in distance is an excellent result. At the lumbar distance, positive effects were also obtained after the first treatment, which was expected because the greatest emphasis was on the central part of the trunk. According to the 2D analysis protocol, Fröner PI did not show statistically significant treatment effects. However, by analyzing the effects according to the KIL-scheme, statistically, significant changes were achieved in the variable Cervico-lumbar lead angle. This variable describes the inclination. Ideal values for inclination are in a range from -2.0-1.0. In the EG, there was a shift from -2.42 to -1.22, approaching the average values from acceptable to ideal, physiological inclination. Through previous research according to the KIL-scheme, it is possible to identify the effects after the application of treatment [5].

**Conclusions**

This study concludes that it is possible to improve postural imbalances after 6-weeks in healthy adolescents young adults. The importance of the research is reflected in the wide application of the proposed CT in children, adolescents, young adults and recreationists, but also with people who are not involved in physical activity by applying exercises that can maintain their working ability.

**References**

1. Karacaoğlu S, Kayapinar FÇ. The effect of core training on posture. Acad. J. Interdiscip. Stud. 2015;4(1S2):221-221.

2. McGill SM, Karpowicz A. Exercises for spine stabilization: motion/motor patterns, stability progressions, and clinical technique. Arch. Phys. Med. Rehabil. 2009:90(1):118-126.

3. Šćepanović T, Marinković D, Korovljev D, Madić D. Status kičmenog stuba u sagitalnoj ravni kod devojčica. In: Proceedings of the 5^th^ International Scientific Conference “Contemporary Kinesiology”-Split. 2015.

4. Kuo YL, Tully EA, Galea MP. Video analysis of sagittal spinal posture in healthy young and older adults. J. Manipulative Physiol. Ther. 2009;32(3):210-215.

5. Mahlknecht J. Das KIL-Schema zur qualitativen und quantitativen Beurteilung der Körperhaltung im seitlichen Profil. Z. Orthop. Ihre Grenzgeb. 2002:140(06):615-620.

## A18 Change of direction speed in volleyball

### Sunčica Poček, Zoran Milosevic, Dusko Cvijovic

#### Faculty of Sport and Physical Education, University of Novi Sad, Novi Sad, Serbia

##### **Correspondence:** Sunčica Poček (suncicapocekfsfv@gmail.com)

**Background**

Volleyball is a team sport in which elite volleyball players possess high level of technical proficiency that allows player to be fully efficient in game settings in terms of kinanthropometric profile potential, e.g., motor abilities. Speed of a hitten ball, size of a court and limited ball contact are challenging for a player to perform basic skills in a constantly changing environment. In a limited time, player should be able to perceive key actions on the court, make a decision and act in a quick and agile manner. In order to do so, there is the need for a rapid whole-body movement with change of velocity or direction in response to a stimulus [1]. Volleyball involve some straight sprinting of about 5 and 10 m distances, rarely longer, but more often repeated short sprinting with changes of direction. The total distance covered by a player during a top-level volleyball match depends on factors such as the number of played sets, a players’ function on the court, area of the court, set scores, the number and duration of individual rallies, and number of type of actions in a rally [2]. Whether it's about pre-planned [3] or reactive activity [4] both change of direction speed and perceptual and decision-making factors which are contained in agility [1] are of great importance in volleyball [5]. Of particular interest in this study is the change of direction (COD) component of agility.

**Methods**

A total of 45 young female volleyball players (table 1) participated in this study. The sample of subjects was divided in three groups by performance quality of 1^st^ (N=15), 2^nd^ (N=10) and 3^rd^ team (N=20). All subjects were assessed for height, weight, and evaluated with a COD speed tests, namely T – test, Sprint 93639 m forward – backward and Sprint 93639 m with 180^0^ turns [6] and Japan test. The study was conducted according to the Helsinki declaration, and ethical approval was obtained from the ethics committee of the University of Novi Sad, Serbia. Statistical analysis was conducted using IBM SPSS Statistics for Windows, Version 20.0.

**Results**

Based on the results of MANOVA (table 1) significant differences were observed between young female volleyball players in terms of age, body height, and COD speed (p≤0.05). Volleyball players from 1^st^ team were statistically different from 2^nd^ a 3^rd^ team, with better results on the COD speed tests and greater value of body height in comparison to 3^rd^ team. There were no statistically significant differences between 2^nd^ and 3^rd^ team in terms of COD speed tests, although volleyball players of the 2^nd^ team were older, with greater body height and selected to team of higher skill proficiency.

One of the main limitations of the CODS tests is that although they may simulate the “general” movement patterns of match-play they don’t offer a valid method of discriminating higher and lesser skilled players [7] in contrast to reactive agility tests. Findings of our study indicated that the greater the difference between performance level quality in terms of skill proficiency of volleyball players the greater the chance of displaying differences in terms of COD speed. Volleyball is a team sport with highly specific tasks and responsibilities for each player on the court according to player’s position [5]. In no other team sport there is as much interdependence of players in scoring as is the case in volleyball. By performing their tasks through the organization of attack, defence, and transition in between, most of the time, they are performing skills through reactive agility. Every skill, besides serve, in volleyball is not only reactive than even more proactive where is always a need in situational efficacy or game setting to implement perceptual and decision-making factors while performing change of direction speed. Training and testing of change of direction speed as a component of agility have a great importance in volleyball [7].

**Conclusions**

Having in mind energetic requirements, type of force application and number of change of directions, from a great deal of variety in the type and number of assessments that have been used to assess COD ability it is proposed that T test, Sprint 93639 m fwdbwd, Sprint 93639 m with 180^0^ turns and Japan test are the best to use in volleyball.

**References**

1. Sheppard JM, Young, WB. Agility literature review: Classifications, training and testing. J. Sport Sci. 2006;24:919-932.

2. Mroczek D, Januszkiewicz A, Kawczynski AS, Borysiuk Z, Chmura, J. Analysis of male volleyball players' motor activities during a top level match. J. Strength Cond. Res. 2014;28:2297-2305.

3. Brughelli M, Cronin J, Levin G, Chaouachi, A. Understanding change of direction ability in sport. Sports Med. 2008;38:1045-1063.

4. Paul DJ, Gabbett TJ, Nassis, GP. Agility in team sports: Testing, training and factors affecting performance. Sports Med. 2016;46:421-442.

5. Pocek S, Vukovic J, Jaksic D, Lakicevic N, Messina G, Bianco A, Drid P. Fitness profile of young female volleyball players. Med. Sport. 2020;73(2):197-209.

6. Sekulic D, Spasic M, Mirkov D, Cavar M, Sattler T. Gender-specific influences of balance, speed, and power on agility performance. J. Strength Cond. Res. 2013;27(3):802-811.

7. Barnes JL, Schilling BK., Falvo MJ, Weiss LW, Creasy AK, Fry AC. Relationship of jumping and agility performance in female volleyball athletes. J. Strength Cond. Res. 2007;21(4):1192-1196.

**Table 1 (abstract A18).** Differences in change of direction speed tests between three groups of young female volleyball players

	1^st^ team	2^nd^ team	3^rd^ team	f	p	partη^2^
Age (yrs.)	18.23±1.71^2,3^	16.63±0.87^1.3^	14.38±0.56^1,2^	50.76	**0.00**	0.71
Height (m)	1.79±0.04^3^	1.78±0.06^3^	1.74±0.05^1.2^	5.95	**0.01**	0.22
Weight (kg)	65.2±5.6	59.3±8.1	61.0±12.5	1.27	0.29	0.06
T test (s)	11.8±0.5^2.3^	12.6±0.9^1^	12.6±0.9^1^	11.21	**0.00**	0.35
Japan test (s)	8.6±0.4^2,3^	9.2±0.8	9.1±0.7	3.80	**0.03**	0.15
93639 fb (s)	8.9±0.4^2,3^	9.8±0.7^1^	9.5±0.5^1^	8.28	**0.00**	0.28
93639 180^0^ (s)	9.4±0.5^2,3^	10.0±0.9^1^	9.9±0.5^1^	4.36	**0.02**	0.17
	F= 8.25 P= **0.00** Partη^2^= 0.62					

93639 fb – test 93639 m forward backward; 93639 180^0^ – test 93639 m with 180^0^ turns

## A19 The influence of anthropometric characteristics on running speed in preschool children

### Nikola Radulović^1^, Ilona Mihajlović^1^, Ratko Pavlović^2^, Mila Vukadinović Jurišić^1^, Jelena Obradović^1^

#### ^1^Faculty of Sport and Physical Education, University of Novi Sad, Novi Sad, Serbia; ^2^Faculty of Physical Education and Sport, University of East Sarajevo, East Sarajevo, Bosnia and Herzegovina

##### **Correspondence:** Nikola Radulović (nikolaradulovicfsfv@gmail.com)

**Background**

Children between the ages of 5−6 look forward to do movements as running, jumping and bouncing by nature and they have such an energy that they cannot stand still [1]. Their coordination abilities are highly developed compared to other age groups and they have the ability to combine two or more movements, as running. Although running speed, reaction time and quick footwork are continuously improving from the age of five to maturity, it is recommended that pre-schoolers work on their speed only when they adopt a proper running technique [2]. Anthropometric measurements can be indicators of a population’s health status, quality of nutrition and nutritional status, giving rise to increasingly widespread studies of anthropometric characteristics among researchers [3] not only for monitoring children’s growth and development, but also for an early detection of overweight and risk of obesity. It is known that there is a correlation between motor abilities and morphological characteristics as confirmed by many studies [4,5], but research on the influence of anthropometric characteristics on running speed at this age are in deficit.

Therefore, the aim of this study was to determine the influence of anthropometric characteristic on running speed in preschool children.

**Methods**

The study was conducted on a sample of 50 male preschool children (aged 6-7 years). At the time of the research, all the respondents were attending the preschool institution "Maslačak" in Sjenica. The study was conducted in mid-October 2019 in the premises of the sports hall in Sjenica. The predictor system of morphological characteristics defined the following anthropometric measures: Body height (cm), Body mass (kg), Extended upper arm circumference (mm), Extended forearm circumference (mm) and Chest circumference (mm). The criterion variable of motor space was defined using the following motor test: Running speed at 20 m standing start run (1/10 s) - to estimate the running speed. In order to determine the effect of the system of predictor variables on the criterion variable, as well as the individual contribution of the predictors to the definition of the criterion variable, Linear Regression Analysis was applied. Statistical analyses were performed with SPSS software (Version 20.0; IBM SPSS, Inc., Chicago, IL, USA).

**Results**

By inspecting Table 1, it can be concluded that there is a statistically significant influence of the predictor system of variables on the criterion variable of Standing start 20 m run, at the multiple correlation value (P = 0.01). The value of the multiple correlation coefficient is high and the system is explained by 52% of the variability of the criterion variable. The remaining percentage of variance (48%) can be attributed to other characteristics and abilities that were not the subject of the given predictor system (other morphological characteristics (skin folds), conative characteristics, cognitive abilities). Observing the individual influence of each predictor variable, it can be concluded that only the variable for assessing the chest circumference has shown a statistically significant influence on the criterion (pbete = 0.02). The higher values of the boys' chest circumference were, the slower the running speed was. Considering the values of the beta coefficient of the Body mass variable, it can be also assumed that the increased body mass had a similar effect on the criterion variable. In addition, the high and negative beta coefficient of the Body height variable has indicated that the boys of higher body height and lower weight and circular dimensionality, achieved better results in 20 m running.

The results of the present study have indicated that there is only a statistically significant influence of the system of predictor variables on the criterion variable of *Standing start 20m run* in boys (P=0.01) where the highest percentage of common variability can be also noticed (52%). Only the predictor variable of Chest circumference is statistically significantly related to the criterion, as in the study of Bala et al [5], which leads us to the conclusion that the preschool boys with higher values of the chest circumference achieved worse results because of their bigger body build, and perhaps because they are taller, which affected their lower performance in the criterion variable considered. Milanese et al. [6] considered when subjects were stratified by age class and sex, males aged 6-7 years only showed a negative correlation between sum of skinfolds and running speed. It is generally known that boys are somewhat faster at preschool age and over time they tend to become even faster than girls. Also, it should be added that in studies of pre-school children these should be classified as per 6 months of age due to the trend of development of morphological characteristics that are different in boys and in girls [7]. The result obtained in the variable of *Standing start 20 m run* was better in the respondents with higher values of body height, lower values of body mass, extended upper arm circumference, extended forearm circumference, than in the subjects with lower values of the specified morphological characteristics. This result has shown that the taller boys of slender figures achieved better results in running speed. Body weight without a certain body height indicates a very stocky build that is negatively correlated with running speed.

**Conclusions**

The results of the study have confirmed the results of the studies of authors on the influence of anthropometric characteristics on motor abilities in this age [6]. In further research, it is necessary to include skinfolds in the equation, if applicable, as well as body composition, in order to obtain more credible and accurate research results.

**References**

1. Gozel Tepe Z. Determining the Motor Ability Levels of the Preschool Children. *J. Educ. Learn.* 2020:9(3);73-78.

2. Stupar DM, Fratrić FF, Nešić M, Rubin P, Međedovic B. The effects of an experimental program of speed development on preschool children. *FU Phys. Ed. Sport.* 2015;139-148.

3. Cadenas-Sánchez C, Artero EG, Concha F, Leyton B, Kain J. Anthropometric characteristics and physical fitness level in relation to body weight status in Chilean preschool children. Nutr. Hosp. 2015;32(1):346-353

4. Radulović N, Mihajlović I, Gušić M, Pavlović R. The effects of volume and skinfolds on sprinter speed in 11-12-year old children. Acta Kinesiol. 2016;56-62.

5. Bala G, Jakšić D, Katić R. Trend of relations between morphological characteristics and motor abilities in preschool children. Coll. Antropol. 2009;33(2):373-385.

6. Milanese C, Bortolami O, Bertucco M, Verlato G, Zancanaro C. Anthropometry and motor fitness in children aged 6-12 years. J. Hum. Sport Exerc. 2010;II:265-279.

7. Pavlica T, Rakic R, Sironjic T. Changes in Morphological Characteristics During the Period 2005-2014 in a Sample of Serbian 7-Year-Old Children. Int. J. Morphol. 2017;35(2):691-697.

**Table 1 (abstract A19).** Results of the regression analysis of *Standing start 20 m run* in the male subsample

Variable	r	p	r_part_	p_part_	Beta	p_bete_
Body height	0.30	0.07	-0.36	0.10	-0.68	0.11
Body mass	0.29	0.08	0.39	0.06	0.48	0.07
Chest circumference	**0.54**	**0.00**	**0.55**	**0.00**	**0.60**	**0.02**
Extended forearm circumference	**0.35**	**0.04**	0.36	0.13	0.87	0.13
Extended upper arm circumference	0.27	0.08	-0.16	0.43	-0.42	0.43
R=0.69 R^2^=0.52 **P=0.01**						

Legend: r – Pearson's correlation coefficient; p – level of statistical significance for r; r_part_ - value of the partial correlation coefficient; p_prat_ - level of statistical significance for r_part_ Beta – regression coefficient; pbete – level of significance of the regression coefficient; R - multiple correlation coefficient; R^2^ -determination coefficient; P – significance of the multiple correlation coefficient

## A20 Effects of different training programs on agility performance of female handball players

### Mila Vukadinović Jurišić, Nikola Radulović, Ilona Mihajlović and Jelena Obradović

#### Faculty of Sport and Physical Education, University of Novi Sad, Novi Sad, Serbia

##### **Correspondence:** Mila Vukadinović Jurišić (mila.vukadinovic88@gmail.com)

**Background**

Handball is a fast game in which players are able to run, change direction, jump and perform handball movement in very short time periods, in accordance with the tactical situation. Michalsik et al [1] noted that the change of direction speed (CODS) and reactive agility (RA) as agility movements are widely used in handball, because of the style of game (high-intensity of the game with fast and explosive changes of direction with or without a ball). Also, the same authors [1] considered that, during a handball match, athletes spend 61.2% of the time throwing, jumping and performing agility movements. The CODS is mainly used during the attack phase in handball game while RA is manifested mainly in defence [2]. High levels of CODS and RA, as well as explosive power and speed enable handball players to perform necessary movements faster, better and more efficiently. The development of aforementioned motor abilities by integration of technical-tactical elements with conditional elements makes the most important part of the preparation of handball players during the pre-competitive period. Thus, small-sided games represent the training method which integrates technical-tactical elements with conditioning elements, with the purpose of improving motor, functional and technical-tactical abilities of team sport athletes. In handball, small-sided games may be an effective method to improve speed, CODS, repeated sprint ability, and aerobic performance [3] while high-intensity interval training can also have a positive effect on handball motor performance [4]. Therefore, the purpose of the study was to compare the effects of different training programs on agility performance of female handball players.

**Methods**

Forty-five young female handball players (who competed in the First National Serbian female handball League for at least 3 years) participated in the study. Subjects were divided into the small-sided games group (SSG; n=15; age 16.13 ± 0.91 years; body height: 1.65 ± 3.24 m; body weight: 62.49 ± 7.07 kg ) and the high-intensity interval training group (HIIT; n=15; age 16.20 ± 0.94; body height: 1.68 ± 6.3 m; body weight: 64.26 ± 4.5 kg) and a control group (CON; n=15; age 16.00 ± 0.93 years; body height: 1.68 ± 4.20 m; body weight: 64.46 ± 6.9 kg). Training programs for all groups lasted for 8 weeks with 4 training sessions per week. Participants in SSG group performed specific trainings (small-sided games) and sport-specific team training (handball training). The small-sided games were organized in four teams (3 vs. 3) excluding goalkeepers, on a playing court with dimension 20m x 20m. Participants in HIIT group performed specific trainings (high-intensity interval training 15s – 15s) and sport-specific team trainings (handball training) for eight weeks. High-intensity interval training is based on two sessions of running at the final speed of the Yo-Yo IRT 1 test (maximal aerobic speed-MAS). The sessions were composed of short 15s intermittent running (velocities ranging from 90-95% MAS) reached at the end of the Yo-Yo IRT 1 test according to Krustrup et al. [5] and 15s of active recovery. Participants in CON group had only sport-specific team training (handball training) for four days a week. Before and after the 8-week interventions participants were tested in change of direction speed (T-test), reactive agility test (RAT) and handball agility specific test (HAST). Helsinki declaration and ethical approval was obtained from the ethics committee of the University of Novi Sad, Serbia (Ref. No 46-12-09/2020-1). Statistical analyses were performed with SPSS software (Version 20.0; IBM SPSS, Inc., Chicago, IL, USA).

**Results**

Table 1 show the effects of the different training programs on agility performance. A significant effect of group (SSG vs. HIIT vs. CON) x time (pre- vs. post) interaction was revealed only for RAT rt (p≤0.005).

The present study aimed to compare the effects of different training programs on agility performance of female handball players. The main finding of our study were:1) after eight weeks of different training programs participants for SSG group greater improvement in RAT than participants for HIIT and CON groups; 2) after eight weeks different training programs participants for SSG, HIIT and CON improved CODS but without statistical significant difference between then. To our knowledge, so far, no published articles have examined the effects of these types of training on RA (total time and response movement time) in female handball players. A similar study, but on football players, provided evidence that RAT (total time, decision time and movement response time) can be improved after small-sided games [6]. Small-sided games provide a powerful training stimulus to improve reactive agility (total time) which was entirely to a very large reduction in decision time and change in movement time [7]. Rodriguez-Fernandez et al. [8] considered that the ability to make a quick decision when changing direction of an attacker or the ball is the result of practice during the small-sided games. This finding is confirmed with our results.

**Conclusions**

Both training programs improved result in T-test (SSG=4.3% vs. HIIT=3.1%) and our results are in accordance with those reported by Iacono et al. [3], which indicated improvement in T-test with similar training program (SSG=2.1% vs. HIIT=1.04%), duration of program and intensity, but on male handball players (25.6±0.5 years). All three training programs did not improve results in HAST in female handball players.

**References**

1. Michalsik LB, Madsen K, Aagaard P. Match performance and physiological capacity of female elite team handball players. Int. J. Sports Med. 2014;35(7):595-607.

2. Spasić M, Krolo A, Zenić N, Delextrat A, Sekulić D. Reactive agility performance in handball; development and evaluation of a sport-specific measurement protocol. J. Sports Sci. Med. 2015;14(3):501-506.

3. Iacono AD, Eliakim A., Meckel Y. Improving fitness of elite handball players: small-sided games vs. high-intensity intermittent training. J. Strength Cond. Res. 2015;29(3):835-843.

4. Alonso-Fernández D, Lima-Correa F, Gutierrez-Sánchez Á, Abadía-García de Vicuña O. Effects of a high-intensity interval training protocol based on functional exercises on performance and body composition in handball female players. J. Hum. Sport Exerc. 2017;12(4):1186-1198.

5. Krustrup P, Mohr M, Amstrup T, Rysgaard T, Johansen J, Steensberg A. et al. The Yo-Yo Intermittent Recovery Test: physiological response, reliability, and validity. Med. Sci. Sports Exerc. 2003;35(4):697-705.

6. Chaouchi A, Chtara M, Hammami R, Chtara H, Turki O, Castagna C. Multidirectional sprints and small-sided games training effect on agility and change of direction agilities in youth soccer. J. Strength Cond. Res. 2014;28(11):3121-3127.

7. Young W & Rogers N. Effects of small-sided game and change-of-direction training on reactive agility and change-of-direction speed. J. Sports Sci. 2014;32(4):307-314.

8. Rodriguez-Fernandez A, Sánchez SJ, Rodriguez-Marroyo JA, Casamichana D, Villa JG. Effects of 5-week pre-season small-sided-game-based training on repeat sprint ability. J. Sports Med. Phys Fitness. 2017;57(5):529-536.

**Table 1 (abstract A20).** Effects of different training programs on agility performance

Variables	Group	Pre-test	Post-test	%	Interaction
	SSG	11.40 (0.87)	10.91 (0.83)*	-4.3	
T-test (s)	HIIT	11.16 (0.64)	10.81 (0.62)*	-3.1	p=0.061, η^2^_p_=0.122
	CON	11.15 (0.71)	11.01 (0.80)	-1.3	
	SSG	2.66 (0.14)	2.48 (0.13)*	-6.8	
RAT tt (s)	HIIT	2.55 (0.13)	2.46 (0.10)*	-3.5	p=0.162, η^2^_p_=0.081
	CON	2.60 (0.14)	2.50 (0.13)*	-3.8	
	SSG	1.64 (0.14)	1.56 (0.10)*a	-4.9	
RAT rt (s)	HIIT	1.62 (0.07)	1.58 (0.08)*	-2.5	**p=0.000**, η^2^_p_=0.232
	CON	1.67 (0.03)	1.64 (0.05)*	-1.8	
	SSG	8.05 (0.68)	7.89 (0.82)	2.0	
HAST (s)	HIIT	7.64(1.00)	7.69(0.90)	0.7	p=0.917, η^2^_p_=0.008
	CON	8.14(0.49)	8.21(0.61)	0.9	

**Abbreviations:** SSG – small-sided games training group; HIIT – height-intensity interval training group; CON – control group; T-test – CODS; RAT tt – reactive agility test with live tester, total time; RAT rt – reactive agility test with live tester, response movement time; % – Pre-test and Post-test changes; p-value - significant difference of p ≤ 0.005; ŋ2_p_ - Partial Eta Squared; * significant Pre- to Post testing at p ≤ 0.005 (the main effect of time); **a** – significant difference between effects SSG and HIIT groups

